# Translational imaging of the fibroblast activation protein (FAP) using the new ligand [^68^Ga]Ga-OncoFAP-DOTAGA

**DOI:** 10.1007/s00259-021-05653-0

**Published:** 2021-12-27

**Authors:** P. Backhaus, F. Gierse, M. C. Burg, F. Büther, I. Asmus, P. Dorten, J. Cufe, W. Roll, D. Neri, S. Cazzamalli, J. Millul, J. Mock, A. Galbiati, A. Zana, K. P. Schäfers, S. Hermann, M. Weckesser, J. Tio, S. Wagner, H.-J. Breyholz, M. Schäfers

**Affiliations:** 1 Department of Nuclear Medicine, University Hospital Münster, Albert-Schweitzer Campus 1, Building A1, 48149 Münster, Germany; 2 European Institute for Molecular Imaging, University of Münster, Waldeyerstraße 15, 48149 Münster, Germany; 3 Clinic for Radiology, University Hospital Münster, Albert-Schweitzer Campus 1, Building A1, 48149 Münster, Germany; 4 Department of Chemistry and Applied Biosciences, Swiss Federal Institute of Technology (ETH Zürich), Vladimir-Prelog-Weg 4, CH-8093 Zurich, Switzerland; 5 Philogen SpA, Piazza La Lizza 7, 53100 Siena, Italy; 6 Philochem AG, Libernstrasse 3, CH-8112 Otelfingen, Switzerland; 7 Department of Gynecology & Obstetrics, University Hospital Münster, Albert-Schweitzer Campus 1, Building A1, 48149 Münster, Germany

**Keywords:** FAPI, PET/CT, PET/MRI, Breast cancer, Molecular imaging, Radiosynthesis

## Abstract

**Purpose:**

The fibroblast activation protein (FAP) is an emerging target for molecular imaging and therapy in cancer. OncoFAP is a novel small organic ligand for FAP with very high affinity. In this translational study, we establish [^68^Ga]Ga-OncoFAP-DOTAGA (^68^Ga-OncoFAP) radiolabeling, benchmark its properties in preclinical imaging, and evaluate its application in clinical PET scanning.

**Methods:**

^68^Ga-OncoFAP was synthesized in a cassette-based fully automated labeling module. Lipophilicity, affinity, and serum stability of ^68^Ga-OncoFAP were assessed by determining log*D*_7.4_, IC_50_ values, and radiochemical purity. ^68^Ga-OncoFAP tumor uptake and imaging properties were assessed in preclinical dynamic PET/MRI in murine subcutaneous tumor models. Finally, biodistribution and uptake in a variety of tumor types were analyzed in 12 patients based on individual clinical indications that received 163 ± 50 MBq ^68^Ga-OncoFAP combined with PET/CT and PET/MRI.

**Results:**

^68^Ga-OncoFAP radiosynthesis was accomplished with high radiochemical yields. Affinity for FAP, lipophilicity, and stability of ^68^Ga-OncoFAP measured are ideally suited for PET imaging. PET and gamma counting–based biodistribution demonstrated beneficial tracer kinetics and high uptake in murine FAP-expressing tumor models with high tumor-to-blood ratios of 8.6 ± 5.1 at 1 h and 38.1 ± 33.1 at 3 h p.i. Clinical ^68^Ga-OncoFAP-PET/CT and PET/MRI demonstrated favorable biodistribution and kinetics with high and reliable uptake in primary cancers (SUV_max_ 12.3 ± 2.3), lymph nodes (SUV_max_ 9.7 ± 8.3), and distant metastases (SUV_max_ up to 20.0).

**Conclusion:**

Favorable radiochemical properties, rapid clearance from organs and soft tissues, and intense tumor uptake validate ^68^Ga-OncoFAP as a powerful alternative to currently available FAP tracers.

**Supplementary Information:**

The online version contains supplementary material available at 10.1007/s00259-021-05653-0.

## Introduction

The fibroblast activation protein (FAP) is a membrane serine protease expressed by fibroblasts. FAP has recently emerged as one of the most promising target structures for molecular imaging and therapy in cancer [1, 2]. In contrast to the ubiquitous presence of non-FAP-expressing quiescent fibroblasts, FAP expression by activated fibroblasts in the adult is (with few exceptions) linked to pathologic states such as wound healing, organ fibrosis, and cancer, where it is abundantly expressed by cancer-associated fibroblasts (CAFs) in the tumor microenvironment [3].

Radiolabeled FAP-targeting small ligands based on FAP inhibitors (FAPI) have recently been introduced and demonstrated very promising characteristics for PET imaging such as high and fast uptake in a variety of cancers and rapid clearance from the majority of healthy organs [4–6]. As of now, a growing body of clinical studies has established a high versatility of FAPI-PET to detect the spread of a wide range of cancers. Importantly, FAPI-PET seems to be able to fill clinically urging shortcomings of ^18^F-FDG, for example in pancreatic cancer [7], primary liver tumors [8], gastric and bowel cancer [9], and breast cancer [10].

In addition to its role in diagnostic imaging, reliable expression in cancer and wide absence in other tissues potentially qualify FAP as a theranostic molecular target. Radioligand therapy using α- or β-emitting isotopes appears tempting, in light of recent break through clinical studies of theranostic agents targeting somatostatin receptors in neuroendocrine tumors [11] and prostate-specific membrane antigen (PSMA) in prostate cancer [12]. However, first published retrospective studies on clinical radioligand therapy with FAPI agents leave open questions regarding their therapeutic efficacy [13, 14]. In addition, targeted delivery of non-radioactive drugs and chimeric antigen receptor (CAR) T-cells has been discussed to employ FAP for targeted molecular therapy [15]. For all of such therapeutic approaches, FAP-targeted molecular imaging will play an indispensable role to stratify patients.

Different specific FAP radioligands have been introduced, of which the ligands initially developed at the University of Heidelberg, i.e., FAPI-04 [6] and FAPI-46 [16], are most abundantly and very successfully used in published PET imaging studies. Recently, the new very high-affinity FAP ligand OncoFAP was shown to possess selective FAP binding in vitro and striking tumor uptake in animal models after ^177^Lu or fluorophore labeling [15]. In this translational study, we describe the radiosynthesis of [^68^Ga]Ga-OncoFAP-DOTAGA (^68^Ga-OncoFAP), its preclinical evaluation in murine tumor models, and results from first clinical application of ^68^Ga-OncoFAP in different cancers.

## Material and methods

### Radiosynthesis, in vitro tests, and assays

^68^Ga-FAPI-46 was synthesized as described previously [10]. FAPI-46 precursor for preclinical and clinical imaging was kindly provided under a MTA by U. Haberkorn (Heidelberg, Germany). OncoFAP-DOTAGA was synthesized as previously reported [15]. Radiolabeling was performed on the basis of the German Pharmaceuticals Act (AMG §13 (2b)), i.e., magistral preparation. Briefly, radiogallium (*T*_½_ = 68 min, *β*^+^ = 89%, and *EC* = 11%) was automatically eluted with 0.1 M HCl (0.36%) from a 50 mCi (1.85 GBq) ^68^Ge/^68^Ga radionuclide generator (EZAG, Berlin, Germany) without pre-purification of the eluate and transferred into the reaction vessel of the ^68^Ga-radiosynthesis module. Two types of single-use disposable cassette-based modules, either a manual iQS Ga-68 Fluidic Labeling Module (itG, Garching, Germany) or a fully automated labeling module (miniAllinOne, Trasis, Ans, Belgium), containing a pre-heated, buffered OncoFAP-DOTAGA solution, were used for radiolabeling. After incubation for a few minutes at ca. 100 °C, the reaction mixture was loaded onto a SPE cartridge, eluted with EtOH in the product vial, and formulated with additional 0.9% NaCl. For clinical imaging, a full QC was performed for each preparation of ^68^Ga-OncoFAP. All QC parameters (see Suppl. Table 1) were in accordance with the Ph. Eur. standards for ^68^Ga-DOTA-TOC (monograph 2482). For preclinical imaging, a restricted QC was performed and ^68^Ga-OncoFAP or ^68^Ga-FAPI-46 product solutions were subsequently concentrated by rotary evaporation under reduced pressure to remove the ethanol and redissolved in a small volume of physiological saline, suitable for injection into mice.

The metabolic stability of ^68^Ga-OncoFAP was assessed after incubation with human and mouse blood serum at 37 °C by analytical radio-HPLC performed at different time points after incubation (i.e., 30, 60, 90, and 120 min).

The lipophilicity of ^68^Ga-OncoFAP-DOTAGA was determined according to previously described procedures [17].

Enzymatic inhibition activity and selectivity of OncoFAP derivatives was assessed by employing commercially available in vitro fluorescence assays for FAP, dipeptidyl peptidase 8 (DDP8), and prolyl oligo peptidase (POP) (BPS Bioscience, San Diego, CA, US). The fluorogenic substrate (Fluorogenic DPP substrate 1, Ala-Pro-AMC dipeptide, AMC: 7-amino-4-methylcumaryl) was incubated together with recombinant FAP, DDP, or POP/PREP in the presence or absence of test compounds. The enzymatic activity was correlated with the amount of cleaved fluorescent product measured by fluorescence spectroscopy. IC_50_ of each test compound was determined from the curves obtained by plotting fluorescence intensities at different concentrations of the inhibitor.

More detailed information is provided in the Supplementary Material.

### Animal studies

All experiments were conducted in accordance with the German Law on the Care and Use of Laboratory Animals and approved by the Landesamt für Natur, Umwelt und Verbraucherschutz of North Rhine-Westphalia, Germany.

For tumor-xenograft models, female NMRI nu/nu mice (Janvier, France), 8–9 weeks old, were housed at a constant temperature (22 °C) and relative humidity (40–55%) under a regular light/dark schedule. Food and water were available ad libitum.

Tumor cells (10^6^ cells in 50 μL saline) were implanted subcutaneously in the shoulders of NMRI mice with HT1080 wildtype (FAP^−^) in one shoulder and with stably transfected human FAP-expressing HT1080 cells (FAP^+^) in the contralateral shoulder. Cells were kindly provided by U. Haberkorn, University of Heidelberg [5]. At day 11 after implantation, mice received whole-body PET/MRI (1 T Mediso nanoScan) imaging with list mode PET for 60 min and additional non-contrast-enhanced anatomic, coronal T1-weighted MRI, followed by late static PET/MRI imaging 180 min p.i. Either ^68^Ga-OncoFAP or ^68^Ga-FAPI-46 (*n* = 6 each) was injected into the tail vein with a syringe pump at 1000 μL/min in a volume of 100 μL 30 s after the start of PET. At the next day (day 12 after implantation), the same mice were injected with the respective other FAP-tracer and received identical dynamic PET/MRI for 60 min. Sixty minutes p.i., mice were sacrificed under deep narcosis and organs and body fluids were harvested, weighed, and γ-counted. All involved measuring hardware (dose calibrator ISOMED 2010, automatic γ-counter PERKIN ELMER WALAC 2480, Twilite coincidence detector Swisstrace, PET/MRI) were cross-calibrated for ^68^Ga. Overall, 3 scans were rejected, because of extravasation (day 11 OncoFAP), inability of i.v. access (day 12 FAPI-46), and insufficient radioactivity (day 12 FAPI-46), respectively. The resulting injected amounts of radioactivity for the remaining 11 ^68^Ga-OncoFAP scans and 10 ^68^Ga-FAPI-46 scans were 19.2 ± 4.4 MBq and 18.2 ± 4.8 MBq, respectively.

Dynamic PET scans were reconstructed in time frames of 1 × 30 s, 5 × 12 s, 5 × 60 s, 4 × 300 s, 2 × 600 s, and 1 × 810 s. Frames 12 and 18 and the late scan are referred to as (rounded) 10 min, 1 h, and 3 h throughout the manuscript. Volume of interests (VOI) of tumors and representative organs were defined on anatomic MRI. Resulting tumor volumes for ^68^Ga-OncoFAP scans for FAP^+^ and FAP^−^ tumors were 0.4 ± 0.36 and 0.22 ± 0.15, and for FAPI-46 scans were 0.34 ± 0.20 and 0.2 ± 0.17 (n.s. different between the two tracers).

### Preclinical pharmacokinetic modeling

Before PET/MRI at day 12, p.i. mice received surgery to establish an extracorporeal circulation shunting of the blood from the femoral artery to the tail vein or contralateral femoral vein as described previously [18]. At 40 cm from the femoral artery, the shunt (0.3 mm inner diameter silicon; total extracorporeal volume 56.6 μL) was led through a Twilite coincidence detector. Acquired blood activity curves were calibrated with a separately measured calibration factor for ^68^Ga according to vendor’s instructions. The curves were corrected for delay and dispersion based on a numerical deconvolution, using dispersion kernels derived from measuring step functions at defined pumping speeds. Standard 2-tissue compartmental (2TCM) pharmacokinetic (PK) modeling and Patlak analysis was performed for the triceps muscle, and FAP^−^ and FAP^+^ tumors using PMOD version 3.703 (PMOD Technologies LLC) and was based on mean PET VOI time activity curves and Twilite-based extracorporeal arterial input functions (AIF). AIF plasma fraction was calculated based on direct hematocrit measurements after imaging (StatStrip® Hb/Hct, Nova Biomedical).

### Patients

We retrospectively analyzed ^68^Ga-OncoFAP-PET/CT and PET/MRI scans of 12 patients. Primary tumors were breast cancer in 8 patients. The other 4 patients had fibrosarcoma, colon cancer, hepatocellular carcinoma, and an unclear cystic pancreatic tumor, respectively. Patients were referred by their treating oncologist on an individual clinical basis to support initial staging or specific diagnostic challenges in relapsed cancer. All patients gave written informed consent for ^68^Ga-OncoFAP-PET/MRI and/or PET/CT imaging and retrospective scientific analysis. Analysis has been approved by the Ethics Committee of the Medical Association of Westphalia-Lippe and the Medical Faculty of the University of Münster (Az. 2021-408-f-S). This study includes all ^68^Ga-OncoFAP-PET/CT and PET/MRI scans conducted at the University Hospital Münster 01-06/2021. No exclusion criteria were applied.

### Clinical PET/CT and PET/MRI

Patients were injected with 163.3 ± 50 MBq ^68^Ga-OncoFAP. Patient were scanned in supine whole-body PET/CT (mCT, Siemens Healthineers) or PET/MRI (mMR, Siemens Healthineers) ~ 1 h p.i. as described previously [10]. Breast cancer patients additionally received prone breast PET/MRI before the whole-body scan ~ 30 min p.i., and subsequent diagnostic contrast-enhanced CT or MRI was added according to the specific clinical demand; see Table 3 for details. One patient underwent dynamic imaging initiated with tracer injection in PET/CT, consisting of list mode PET of a mediastinal field of view for 2 min (reconstructed frames 6 × 10 s, 3 × 20 s) and subsequent whole-body dynamic scanning consisting of 6 bed positions that were scanned 7 × 45 s from the skull base to mid-thighs. Eventually, 40 min p.i., a whole-body scan with 3-min per bed positions was acquired. Reading of PET/MRI and PET/CT was performed according to a standard clinical workflow. Standard uptake value (SUV) measurements were acquired in syngo.via (Siemens Healthineers) with circular or spherical volumes of interests.

### Statistics

Statistical analysis was performed using MATLAB (R2020a, MathWorks). Mann-Whitney *U* tests were performed for pairwise comparisons. *p* values < .05 were considered statistically significant. If needed, Bonferroni correction was applied to account for multiple testing. All values are displayed as mean ± std if not stated otherwise.

## Results

### Radiosynthesis, in vitro tests, and assays

**Scheme 1:**
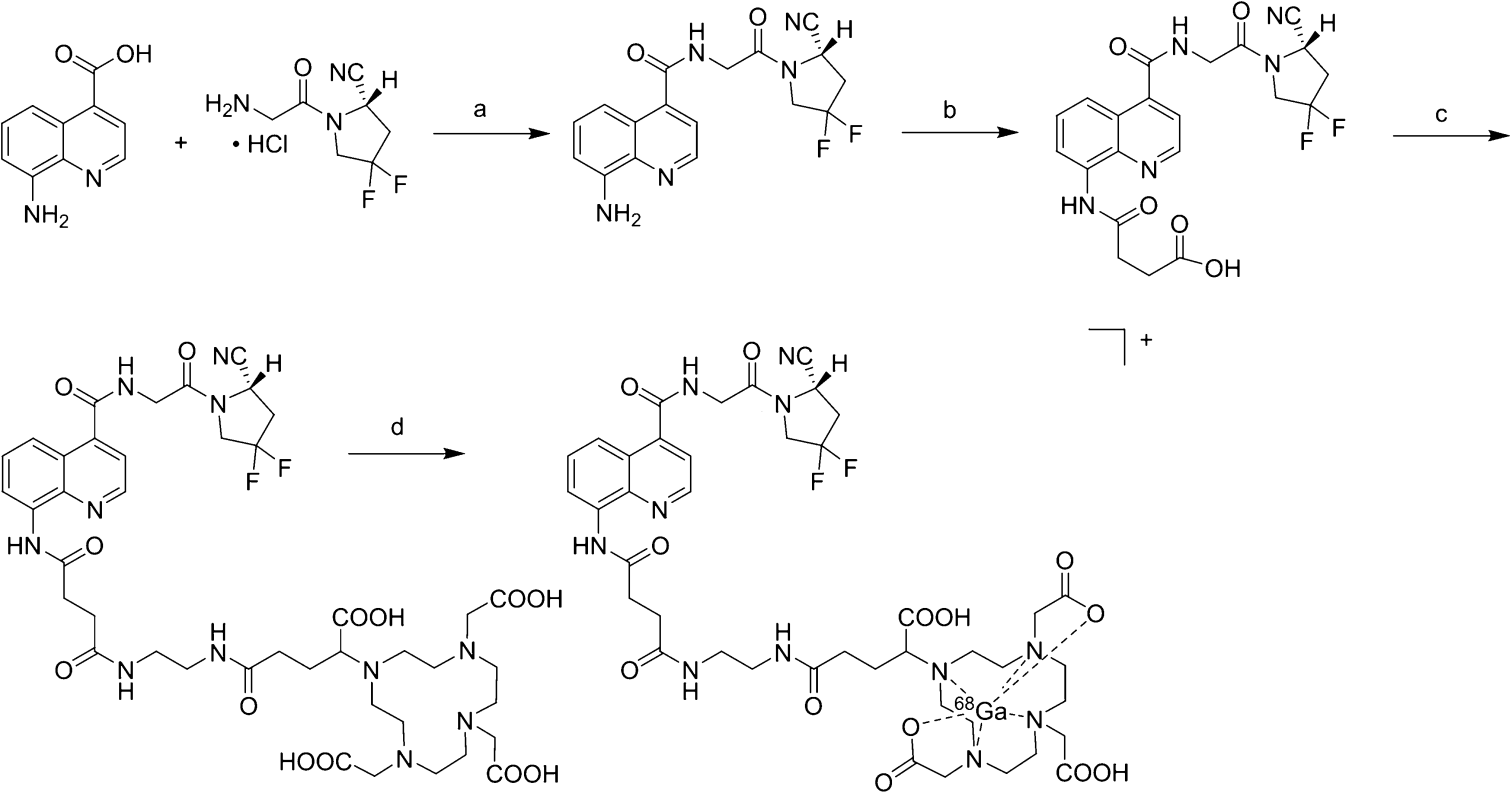
**a**–**c** Organic synthesis as published previously [15] and **d** radiolabelling of OncoFAP-DOTAGA. **a** HATU, DIPEA, DMF/DCM, r. t., 90%. **b** Succinic anhydride, DMAP, THF, 60 °C, 95%. **c** 1. N-Hydroxysuccinimide, DCC, DMSO, r. t.; 2. DOTAGA-NH_2_, PBS, r.t., 10%. **d** [^68^Ga]GaCl_3_, sodium acetate, ascorbic acid, 110–120 °C, 7 min, rcp: 98.1 ± 1.1%, rcy: 75.3 ± 2.9% (*n* = 16, not corrected for decay) for Trasis miniAIO and rcp: 97.5 ± 1.1%, rcy: 69.1 ± 12.7% (*n* = 16, not corrected for decay) for iQS Ga-68 Fluidic Labeling Module

OncoFAP-DOTAGA was synthesized as shown in Scheme 1 [15]. ^68^Ga-OncoFAP was prepared using a manual and a fully automated synthesis module with a radiochemical yield (rcy) of 69.1 ± 12.7% and 75.3 ± 2.9% and radiochemical purity (rcp) of 97.5 ± 1.1% and 98.1 ± 1.1%, respectively (*n* = 16 for each module). All QC parameters were in accordance with the Ph. Eur. standards given for ^68^Ga-DOTA-TOC, and thus, syntheses on both modules were suitable for application in the clinics (for details, see Suppl. Table 1). The non-radioactive tracer analogs [^nat^Ga]Ga-OncoFAP-DOTAGA (^nat^Ga-OncoFAP) and [^nat^Ga]Ga-FAPI-46 (^nat^Ga-FAPI-46) were synthesized as described in the Supplemental Material.

**Fig. 1:**
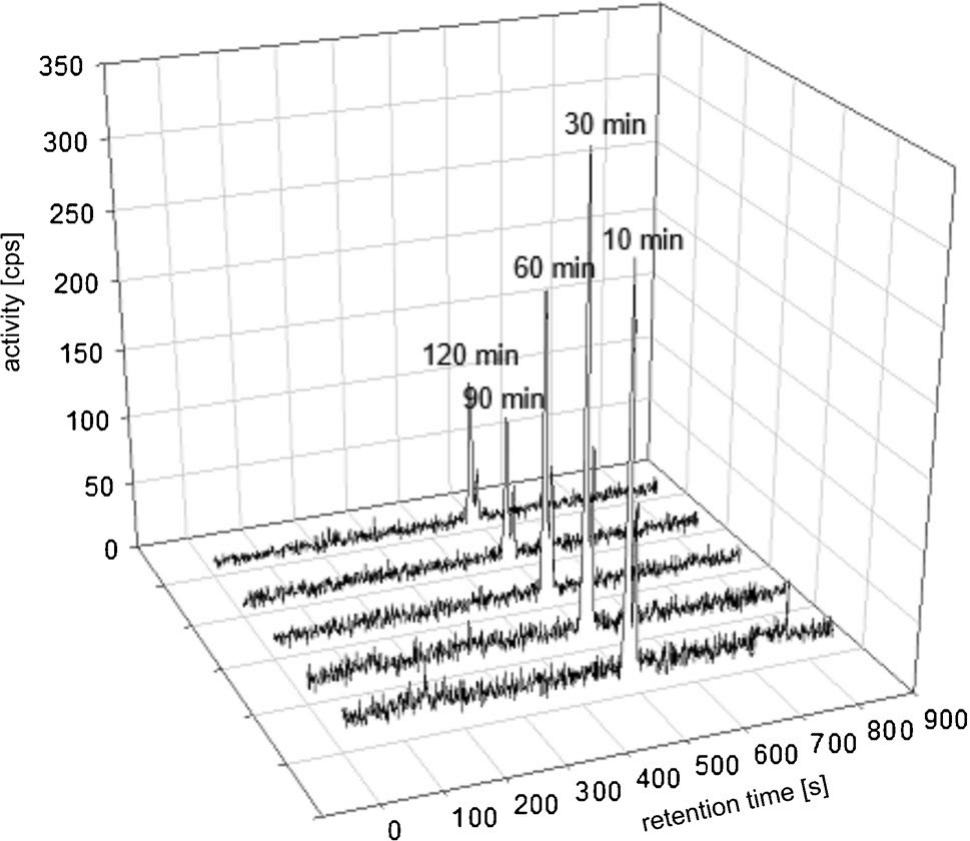
Stability of [^68^Ga]Ga-OncoFAP-DOTAGA in human blood serum at 37 °C as analyzed by analytical radio-HPLC. Radio-HPLC chromatograms are shown for incubation times from 10 to 120 min. See Suppl. Fig. 3 for murine serum stability

Stability of ^68^Ga-OncoFAP was assessed by radio-HPLC chromatograms after incubation in serum (Fig. 1 for human serum, Suppl. Fig. 3 for mouse serum). As a result, ^68^Ga-OncoFAP shows metabolic stability in murine as well as human blood serum for at least 120 min. The experimental log*D*_7.4_ value was − 3.91 ± 0.32 (*n* = 6). Thus, ^68^Ga-OncoFAP can be considered to be highly hydrophilic.

We assessed the enzymatic inhibition activity of ^nat^Ga-OncoFAP to FAP and structurally related members of this family: DPP8 and POP. As reference compounds, the FAPI tracer ^nat^Ga-FAPI-46, the unselective boronic acid–based inhibitor talabostat, and the highly potent POP inhibitor S 17092 (Suppl. Fig. 1) were analyzed. Experimental IC_50_ values are reported in Table 1. Values for talabostat and S 17092 were in accordance with literature [19, 20]. The resulting sub-nanomolar binding affinity to human FAP of ^nat^Ga-OncoFAP (IC_50_ of 0.51 ± 0.11 nM, *n* = 3) was well in line with reported affinity of other derivatives of OncoFAP [15]. There was a high selectivity for FAP compared to DDP8 (1347-fold) and POP (96-fold). As expected, ^nat^Ga-FAPI-46 also displayed high binding potency (IC_50_ of 8.37 ± 0.71 nM, *n* = 3) and selectivity to human FAP compared to DPP8 and POP (> 1000-fold for each). However, our results suggested a superior binding potency (ca. 16-fold) for ^nat^Ga-OncoFAP compared to ^nat^Ga-FAPI-46.

**Table 1:** Inhibition potencies and lipophilicity of ^nat^Ga-OncoFAP compared to selected reference compounds and ^nat^Ga-FAPI-46. Log*D*_7.4_ value were determined using the radiolabeled analog. Literature values [19, 20] are displayed in brackets

Compound	IC_50_ (nM)			Log*D*_7.4_
	FAP	DPP8	POP	
Talabostat	309 ± 56 (560)	92 ± 5 (4)	n. d. (390)	n. d.
S 17092	n. d.	n. d.	6 ± 3 (*K*_i_: 1.5)	n. d.
^nat^Ga-OncoFAP	0.51 ± 0.11	6870 ± 111	49.20 ± 6.30	− 3.91 ± 0.32
^nat^Ga-FAPI-46	8.37 ± 0.71	10.2 ± 3.0 μM	> 10 μM	n. d.

### Biodistribution in a murine cancer model

**Fig. 2:**
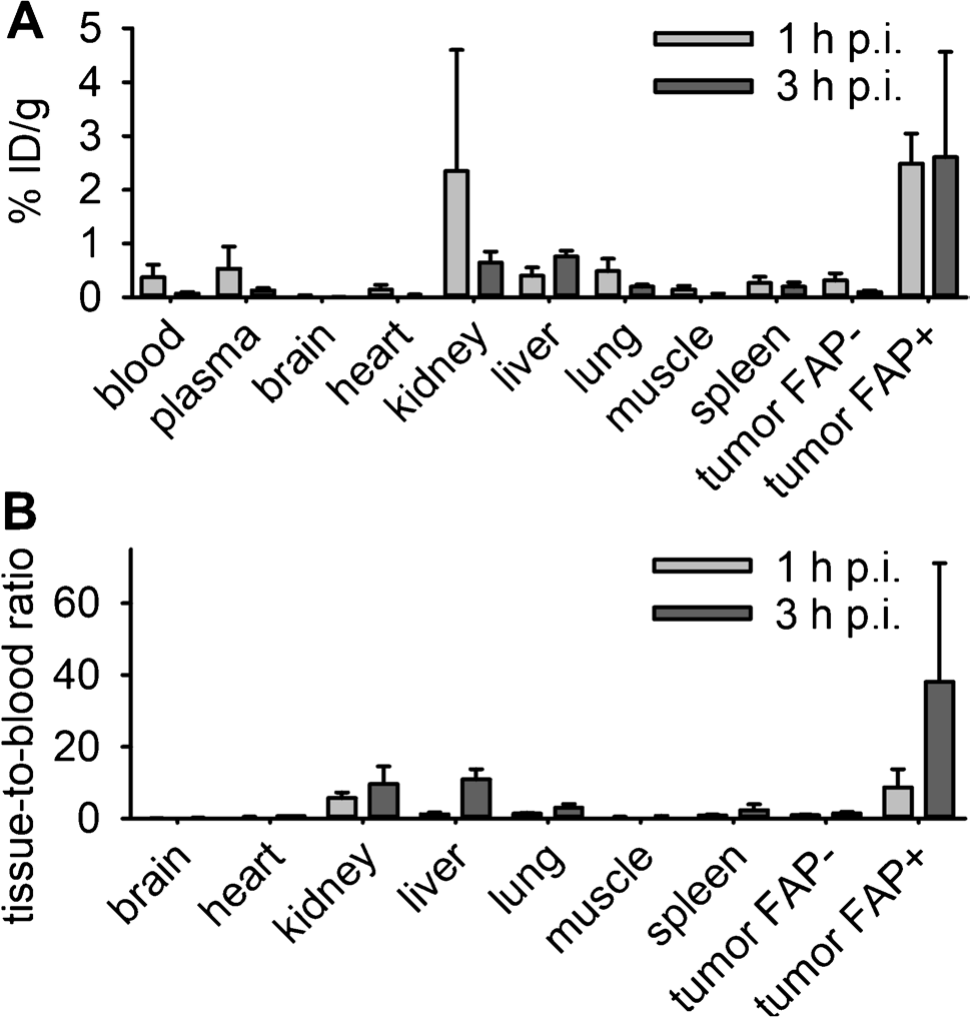
**A** Results from gamma counting in % injected dose per g (% ID/g) of organs harvested 1 h and 3 h after i.v. injection of ^68^Ga-OncoFAP, and **B** tissue-to-blood ratios in mice bearing subcutaneous HT1080 (left shoulder, FAP^−^) and HT1080-hFAP (right shoulder, FAP^+^) tumors (6 mice for each time point). ^68^Ga-OncoFAP demonstrated strong and selective tumor accumulation in FAP^+^ tumors. In contrast to blood and FAP^−^ tumors, accumulation persisted in FAP^+^ tumors from 1 to 3 h resulting in incrementing binding ratios. 3 h p.i. only trace uptake remained in blood and most organs except for the liver, kidney, and spleen. See Suppl. Tables 2–4 and Suppl. Figs. 4 and 5 for all gamma counting data

Biodistribution and uptake in tumors were assessed in mice simultaneously bearing subcutaneous human FAP^+^ and FAP^−^ HT-1080 tumors on the left and right shoulders, respectively (Fig. 2A, B). Gamma counting of harvested organs and tumors 1 h and 3 h after injection of ^68^Ga-OncoFAP demonstrated strong, specific, and temporally stable accumulation in FAP^+^ tumors with increasing contrast due to fast washout from blood (FAP^+^ tumor-to-blood ratio 1 h p.i.: 8.6 ± 5.1 (*n* = 6), 3 h p.i.: 38.1 ± 33.1 (*n* = 6); ratio FAP^+^ tumor/FAP^−^ tumor 1 h p.i.: 9.5 ± 5.6, 3 h p.i.: 25.3 ± 19.2). In contrast to other organs, the liver, kidney and spleen showed elevated retention at late time points (Suppl. Tables 2 and 3). Head-to-head comparison with ^68^Ga-FAPI-46 revealed significantly higher uptake and tissue-to-blood ratios in FAP^+^ tumors for ^68^Ga-OncoFAP 1 h p.i. and comparable uptake between the two tracers 3 h p.i. (uptake in % injected dose per g [% ID/g] 1 h ^68^Ga-OncoFAP: 2.49 ± 0.56 (*n* = 6), ^68^Ga-FAPI-46: 1.28 ± 0.40 (*n* = 4), *p* = .01; tumor-to-blood ratio ^68^Ga-OncoFAP: 8.61 ± 5.1, ^68^Ga-FAPI-46: 1.98 ± 0.92; uptake [% ID/g] 3 h ^68^Ga-OncoFAP: 2.60 ± 1.96 (*n* = 6), ^68^Ga-FAPI-46: 2.64 ± 0.60 (*n* = 6), *p* = .59; tumor-to-blood ratio ^68^Ga-OncoFAP: 38.06 ± 33.08, ^68^Ga-FAPI-46: 28.62 ± 17.69; Suppl. Figs. 4 and 5, Suppl. Tables 2 and 3).

**Fig. 3:**
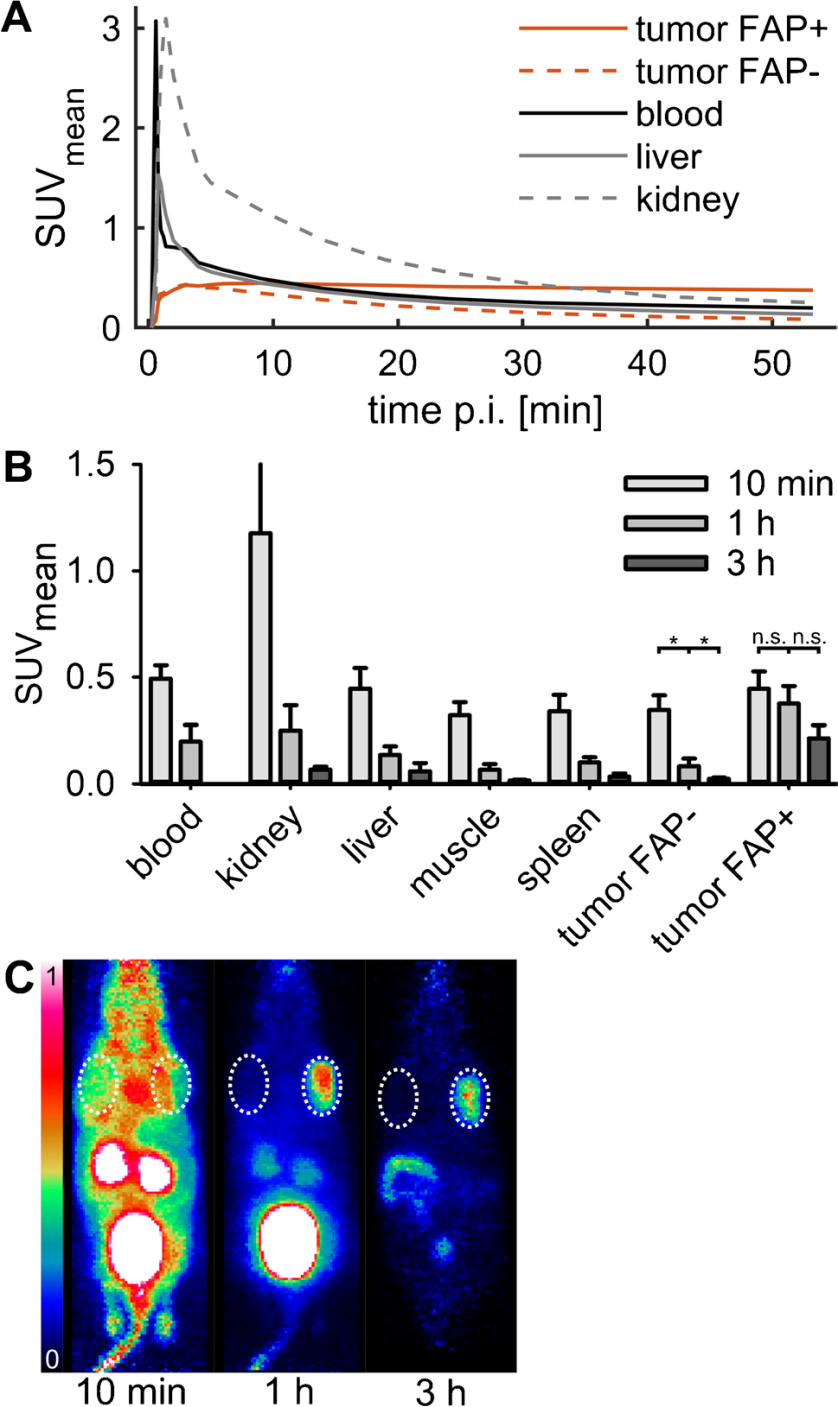
Results from dynamic PET of mice (11 mice, 0–60 min p.i. of ^68^Ga-OncoFAP) bearing subcutaneous HT1080 (left shoulder, FAP^−^) and HT1080-FAP (right shoulder, FAP^+^) tumors. Of these, 5 were additionally measured 3 h p.i. **A** Mean time activity curves of 11 mice. **B** Uptake in organs and tumors 10 min (*n* = 11), 1 h (*n* = 11), and 3 h p.i. (*n* = 5), with significantly different results for FAP-negative tumors (10 min: 0.35 ± 0.07, 1 h: 0.08 ± 0.04, 3 h: 0.02 ± 0.01, 10 min vs 1 h: *p* < .001, 1 h vs 3 h: *p* < .001) and non-significantly different values for FAP-positive tumors (10 min: 0.45 ± 0.08, 1 h: 0.38 ± 0.08, 3 h: 0.21 ± 0.06, 10 min vs 1 h: *p* = .09, 1 h vs 3 h: *p* = .055). The whisker of 10-min kidney activity is truncated to better display the activity range of the remainder of organs/materials. See Suppl. Tables 5 and 6 and Suppl. Figs. 6 and 7 for all PET measurement data. **C** Example of mouse PET maximum intensity projection (MIP) 10 min, 1 h, and 3 h p.i. scaled to SUV 0–1. FAP^−^ (left shoulder, left in image) and FAP^+^ (right shoulder, right in image) tumors are highlighted

In dynamic PET imaging, initial wash in of ^68^Ga-OncoFAP into FAP^+^ and FAP^−^ tumors was comparable (Fig. 3A, B); However, the tracer was rapidly washed out of blood, kidney, liver, muscle, spleen, and FAP^−^ tumors, whereas it was retained in FAP^+^ tumors (Fig. 3C) in agreement with results from gamma counting. Thus, uptake in FAP^+^ and FAP^−^ tumors differed significantly from 10 min p.i. onwards (6 min: *p* = .13, 10 min: *p* = .005, 1 h: *p* < .001 3 h: *p* = .008, *n* = 11 for up to 1 h and n = 5 for 3 h measurements) and the ratio of uptake between FAP^+^ and FAP^−^ tumors grew steadily from 1.0 at 4 min p.i. to 1.3 at 10 min p.i., to 5.0 at 1 h p.i., and to 9.3 at 3 h p.i. The uptake after 1 h at FAP^+^ tumors was significantly higher with ^68^Ga-OncoFAP as compared to ^68^Ga-FAPI-46, but not in delayed scanning after 3 h (SUV_mean_ 1 h, ^68^Ga-OncoFAP: 0.38 ± 0.08 (*n* = 11), ^68^Ga-FAPI46: 0.25 ± 0.06 (*n* = 10), *p* = .004; SUV_mean_ 3 h, ^68^Ga-OncoFAP: 0.21 ± 0.06 (*n* = 5), ^68^Ga-FAPI46: 0.16 ± 0.06 (*n* = 6), *p* = .25; Suppl. Figs. 6 and 7, Suppl. Tables 5 and 6). Noteworthy, all individual mice that were measured with both tracers (*n* = 9) showed higher ^68^Ga-OncoFAP than ^68^Ga-FAPI-46 uptake in FAP^+^ tumors 1 h p.i. independent of the order of measurements (Suppl. Fig. 6 C).

Pharmacokinetic modeling using both the Patlak analysis and a standard two-tissue compartment model were applied based on VOI-derived ^68^Ga-OncoFAP-PET time activity curves of muscle, FAP^−^ and FAP^+^ tumors, and an extracorporeally derived AIF. The Patlak analysis revealed comparable Patlak intercepts between all three tissues tested. However, the Patlak slope as an indicator of specific binding was significantly different between FAP^−^ and FAP^+^ tumors (Table 2). Similarly, transfer constants in pharmacokinetic modeling between plasma and the first compartment (k1 and k2), representing passive transfer from blood into tissue, were similar between the tumors. In contrast, transfer constant fraction k3/k4 and the distribution volume (Vs), representing specific tracer binding, were largely and statistically different. The differences in FAP^+^ tumor uptake comparing ^68^Ga-OncoFAP with ^68^Ga-FAPI-46 were also reflected by significantly different Patlak slopes, k3/k4, and Vs confined to FAP^+^ tumors in between the tracers (geometric mean ± std, Patlak slope ^68^Ga-OncoFAP: 0.007 ± 0.005 (*n* = 6), ^68^Ga-FAPI-46: 0.002 ± 0.001 (*n* = 4), *p* = .02; k3/k4 ^68^Ga-OncoFAP: 7.91 ± 4.83, ^68^Ga-FAPI-46: 1.77 ± 2.74, *p* = .04; Vs ^68^Ga-OncoFAP: 1.34 ± 0.78, ^68^Ga-FAPI-46: 0.25 ± 0.29, *p* = .02; Suppl. Table 7).

**Table 2:** Results of pharmacokinetic modeling of dynamic small animal PET using invasive measurements of the arterial input function (AIF) with an extracorporeal circulation and the Twilite measurement unit. A 2-tissue compartment model (2TCM) (k1–k4) and Patlak modeling were applied. Additionally, the k3/k4 ratio and the distribution volume Vs are calculated for 2TCM. Values are displayed as geometric mean ± SD. k1 and k2 demonstrated only small differences between FAP^−^ and FAP^+^ tumors reflecting similar passive wash in and wash out of tracer. In contrast, k3/k4, and Vs were significantly different between FAP^−^ and FAP^+^ tumors. This finding establishes specific binding of ^68^Ga-OncoFAP-DOTAGA only in FAP^+^ tumors. Consistently, the Patlak model demonstrated comparable Patlak intercept, and a significantly different Patlak slope. No correction of multiple testing was performed due to the strong interdependence of the tested variables. * indicates significance *p* < .05

	Muscle	Tumor FAP^−^	Tumor FAP^+^	*p*-value FAP^−^ vs FAP^+^
k1	0.071 ± 0.018	0.049 ± 0.010	0.040 ± 0.009	
k2	0.471 ± 0.093	0.331 ± 0.046	0.235 ± 0.034	
k3	0.010 ± 0.022	0.015 ± 0.023	0.062 ± 0.015	
k4	0.026 ± 0.063	0.020 ± 0.032	0.008 ± 0.004	
k3/k4	0.383 ± 3.615	0.740 ± 0.825	7.913 ± 4.825	.002*
Vs	0.058 ± 0.485	0.109 ± 0.140	1.336 ± 0.778	.002*
Patlak slope	0.000 ± 0.000	0.000 ± 0.000	0.007 ± 0.005	.002*
Patlak intercept	0.200 ± 0.033	0.216 ± 0.061	0.196 ± 0.074	.59

### First time clinical scanning in patients with various cancers

**Fig. 4:**
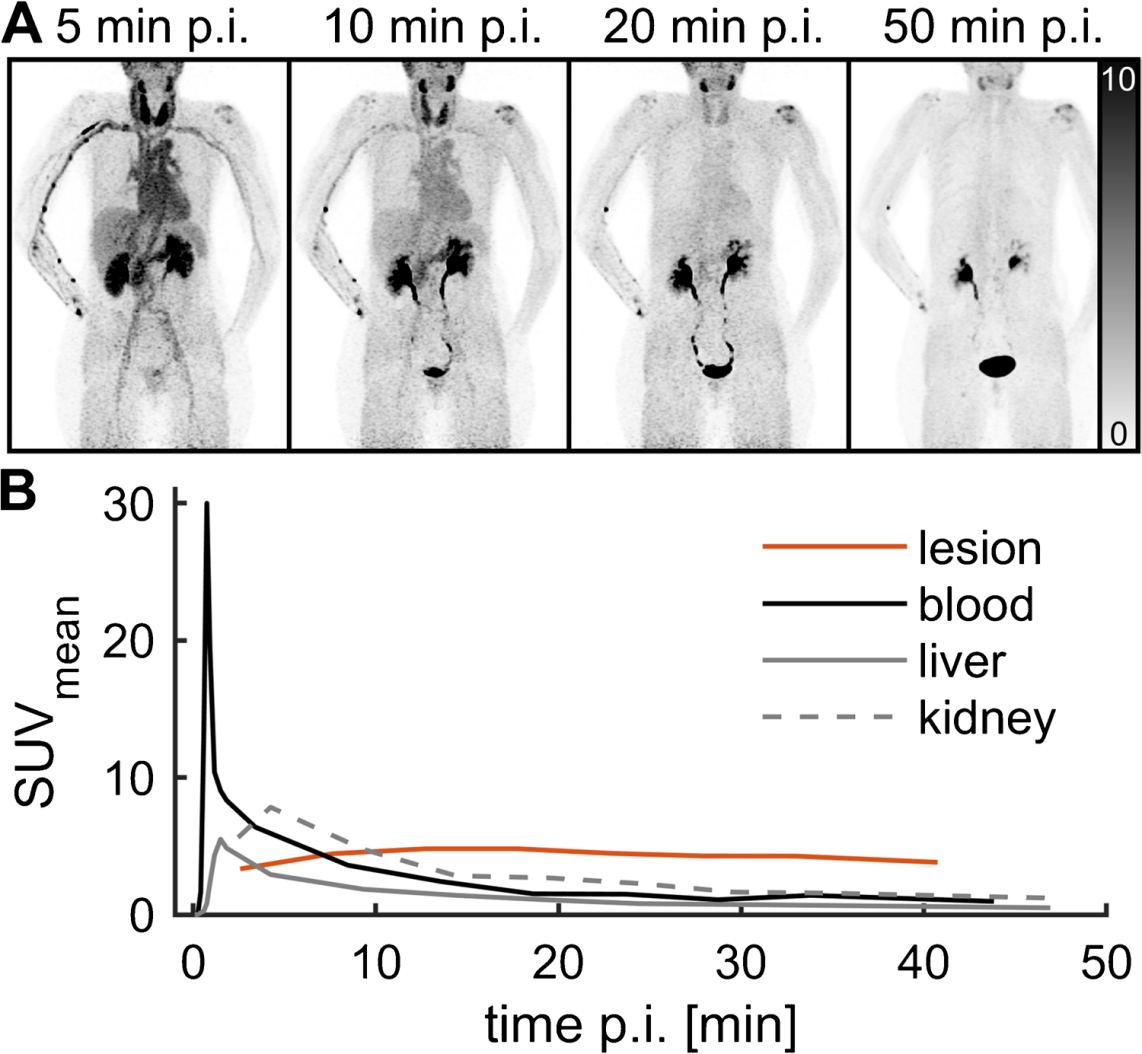
Results from a 60-min whole-body dynamic ^68^Ga-OncoFAP-PET/CT in a female patient. This patient had a history of bilateral breast cancer and was examined for bilateral intermediate axillary lymph node enlargement. Enlarged lymph nodes showed no ^68^Ga-OncoFAP uptake and eventually no recurrence was established. Patient had bursitis of the left shoulder as a secondary finding. The shoulder demonstrated strong tracer uptake that persisted over time, whereas tracer was washed out rapidly from organs. **A** Maximum intensity projections (MIP) of 5, 10, 20, and 50 min p.i. (rounded). MIPs are adjusted to SUV 0–10. **B** Time activity curves (TAC) of VOIs of left shoulder lesion (bursitis), blood, liver, and kidney

Based on successful targeting of FAP in preclinical tumor models, ^68^Ga-OncoFAP was applied in clinical imaging in a total of 12 patients with various cancers based on clinical indications. In one patient, whole-body PET/CT was acquired dynamically over 60 min (Fig. 4). This patient had a history of bilateral breast cancer and was scanned because of equivocal bilateral axillary lymph node enlargement. The whole-body dynamics displayed rapid clearing of the blood pool and organs over the course of 1 h. No uptake was observed in axillary lymph nodes, and eventually, lymph node enlargement was clinically judged as benign taking into account all available imaging (PET/CT, MRI, and ultrasound). By contrast, ^68^Ga-OncoFAP accumulated in a previously diagnosed bursitis of the left shoulder, likely reflecting specific tracer binding in the context of inflammatory tissue remodeling.

**Fig. 5:**
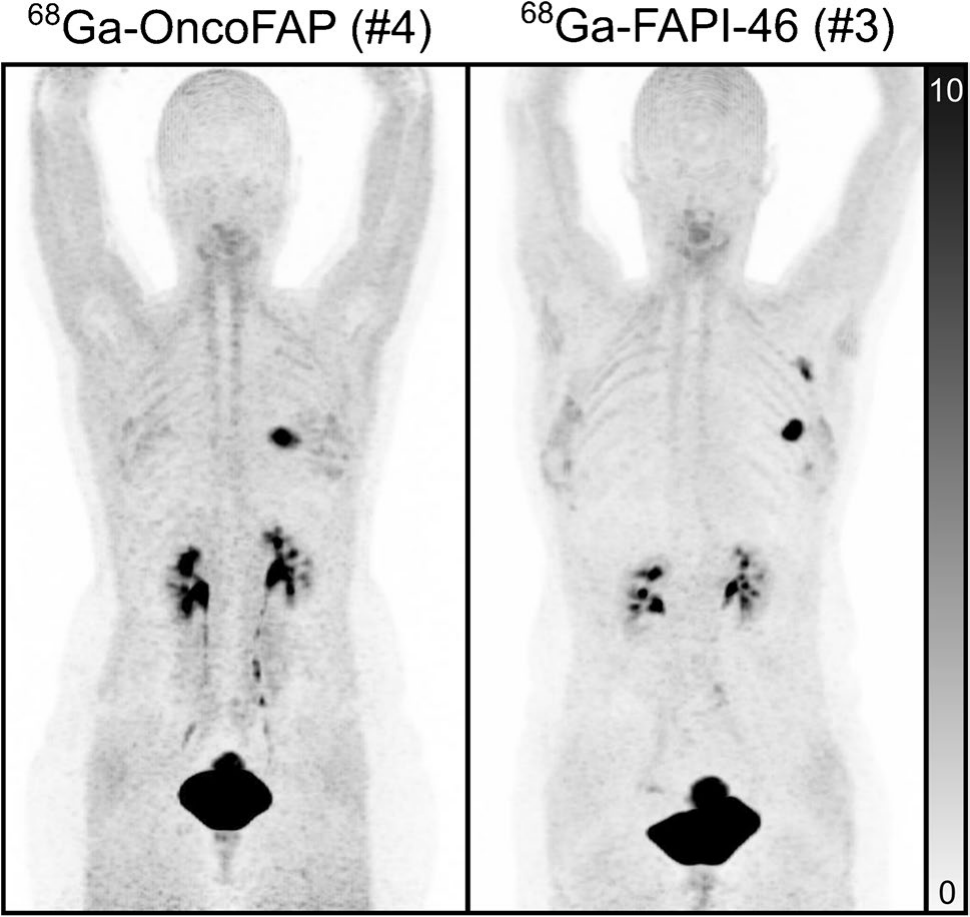
Two examples of breast cancer patients scanned with ^68^Ga-OncoFAP (left, patient #4) and ^68^Ga-FAPI-46 (right, corresponding to patient #3 from [10]). Patient #4 has a left-sided primary breast tumor and no metastases. The patient scanned with ^68^Ga-FAPI-46 also had a left-sided primary breast cancer of comparable size and lymph node metastases. Tracer distribution to organs and soft tissues is highly similar. Muscular uptake was variable for both tracers and is slightly higher in the ^68^Ga-OncoFAP example patient. No significant difference of muscular uptake was observed when comparing the ^68^Ga-OncoFAP and ^68^Ga-FAPI-46 study samples (Table 3)

We compared the biodistribution and tumor binding of ^68^Ga-OncoFAP to a previously published study sample of 19 patients with breast cancer scanned with the FAP ligand ^68^Ga-FAPI-46 (Table 3, Fig. 5) [10]. Patient and acquisition characteristics of the two samples were largely identical except for a higher fraction of males and a broader spectrum of underlying malignancies in the patients scanned with ^68^Ga-OncoFAP. Overall, biodistribution was widely comparable between the two tracers; however, ^68^Ga-OncoFAP showed significantly lower liver uptake (liver SUV_mean_^68^Ga-OncoFAP 0.6 ± 0.2, ^68^Ga-FAPI-46 0.9 ± 0.3, *p* < .003). Highly intense ^68^Ga-OncoFAP and ^68^Ga-FAPI-46 uptake of the uterus was observed in females (OncoFAP: 17.7 ± 6.3 (*n* = 5), FAPI-46: 11.6 ± 4.5 (*n* = 18), *p* = .03), probably reflecting fibroblast activation in cyclically changing tissue and in fibroids.

**Table 3:** Comparison of patient characteristics, acquisition characteristics, SUV_max_ of tumor tissues and other specific uptake sites (e.g., uterus), and SUV_mean_ in blood and non-targeted organs. The study sample of patients scanned with ^68^Ga-OncoFAP is compared to a previously published study sample scanned at our institution with ^68^Ga-FAPI-46. In contrast to the ^68^Ga-OncoFAP study sample, the ^68^Ga-FAPI-46 consisted only of female patients with breast cancer. No other significant differences of patient characteristics and imaging characteristics were found. Slight differences of compared SUV could be found between the two tracers. ^68^Ga-OncoFAP demonstrated higher uptake in the uterus and pancreas, whereas ^68^Ga-FAPI-46 demonstrated higher uptake in the liver and spleen. However, following Bonferroni correction for multiple testing results (*p*-value threshold for significance indicated by * for patient characterics, imaging characteristics and SUV_max_ of targeted lesions of *p* < .013, and for SUV_mean_ in remaining organs of *p* < .006), only the hepatic uptake remained statistically different

Measurement	^68^Ga-OncoFAP mean ± SD (*n* = 12, if not specified)	^68^Ga-FAPI-46 mean ± SD (*n* = 19, if not specified) [10]	*p*-value
Age	52 ± 14	49 ± 9	.41
Female/male	8/4	19/0	.009*
Weight	74.9 ± 12.3	71.2 ± 14.4	.28
Breast cancer/other disease	8/4	19/0	.009*
Whole-body PET/CT/PET/MRI	7/5	9/10	.57
Activity (MBq)	163.3 ± 49.5	148.8 ± 47.9	.60
Supine whole-body imaging (min p.i.)	64 ± 18	79 ± 18	.06
Prone breast imaging (min p.i.)	41 ± 31 (*n* = 7)	29 ± 6 (*n* = 18)	.27
Primary breast cancer (prone SUV_max_ )	12.3 ± 2.3 (*n* = 6)	14.0 ± 5.7 (*n* = 17)	.86
Breast cancer LN metastasis (supine SUV_max_ )	9.7 ± 8.3 (*n* = 5)	12.2 ± 6.2 (*n* = 13)	.39
Distant metastases any cancer (supine SUV_max_ )	14.0 ± 6.7 (*n* = 4)	12.3 ± 0.2 (*n* = 2)	> .99
Uterus (supine SUV_max_)	17.7 ± 6.3 (*n* = 5)	11.6 ± 4.5 (*n* = 18)	.03
Blood (prone SUV_mean_)	1.6 ± 0.3 (*n* = 7)	1.7 ± 0.2	.32
Blood (supine SUV_mean_)	1.2 ± 0.2	1.3 ± 0.3	.19
Muscle (supine SUV_mean_)	1.4 ± 0.5	1.1 ± 0.2	.11
Liver (supine SUV_mean_)	0.6 ± 0.2	0.9 ± 0.3	.003*
Spleen (supine SUV_mean_)	0.8 ± 0.2	1.1 ± 0.8	.02
Pancreas (supine SUV_mean_)	1.7 ± 0.7	1.1 ± 0.4	.03
Bone (supine SUV_mean_)	0.6 ± 0.2	0.4 ± 0.1	.09
Kidney (supine SUV_mean_)	1.5 ± 0.4	1.5 ± 0.4	.72

**Fig. 6:**
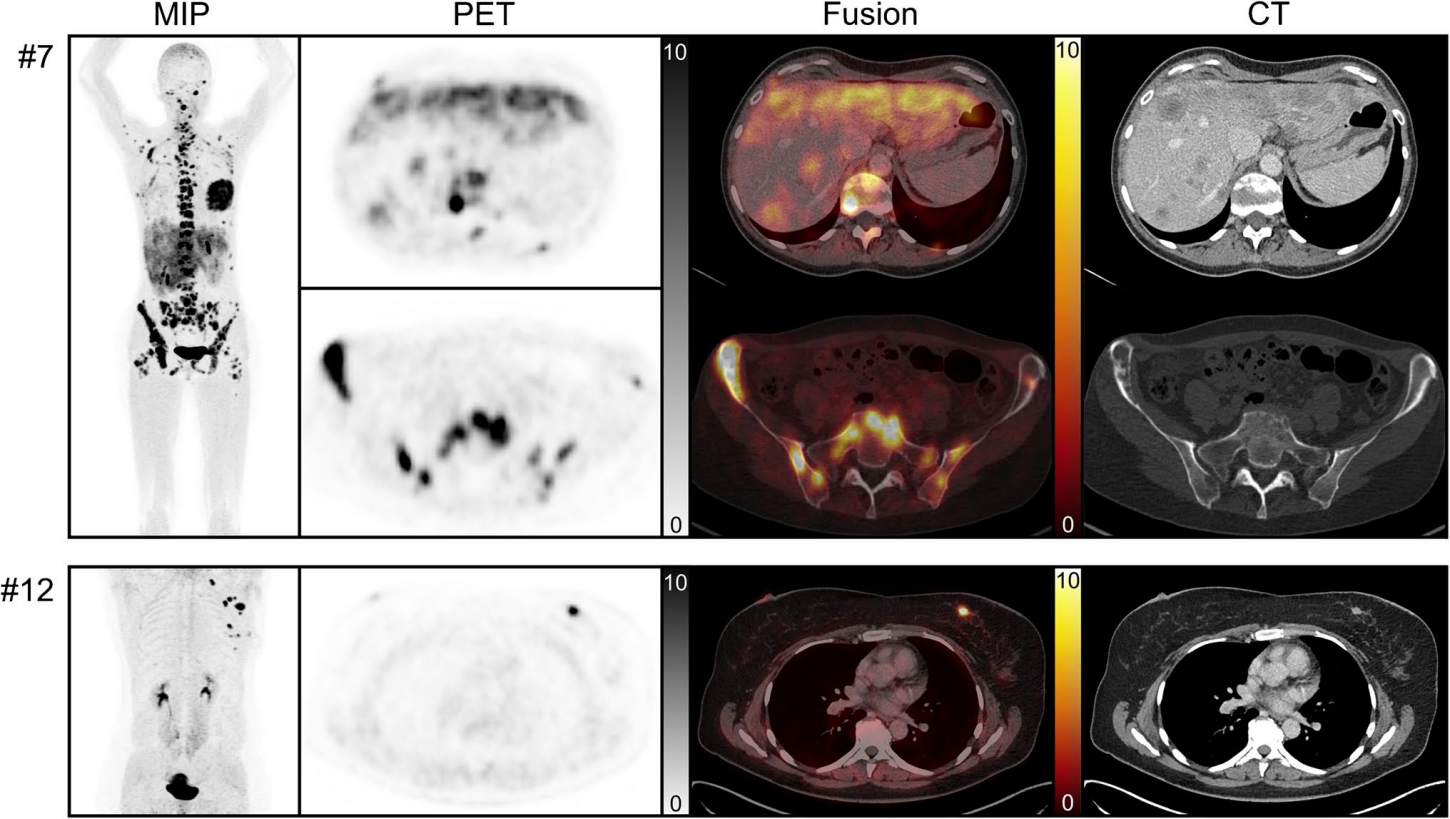
Two examples of patients scanned with ^68^Ga-OncoFAP. Patient #7 with newly widespread metastatic disease to lymph nodes, bones, and liver at primary diagnosis of locally advanced breast cancer. Whole-body maximum intensity projection (MIP), PET, fusion, and contrast-enhanced CT are shown in a slice at the liver and the pelvis demonstrating osseous (SUV_max_ 19.5) and hepatic metastases (SUV_max_ 9.9). Patient #12 with newly diagnosed breast cancer metastatic to local and supraclavicular lymph nodes. MIP, PET, fusion, and contrast-enhanced CT are shown in a slice at the breast demonstrating strong uptake of even sub-centimeter breast lesions (SUV_max_ 15.1). All PET images are adjusted to SUV 0–10

In breast cancer, ^68^Ga-OncoFAP demonstrated highly intense targeting of primary tumors (SUV_max_: 12.3 ± 2.3, *n* = 6), lymph node metastases (SUV_max_ 9.7 ± 8.3, *n* = 5), and distant metastases (up to SUV_max_ 19.5) comparable to previously published data on ^68^Ga-FAPI-46 (Fig. 6, Table 3) [10]. Focal ^68^Ga-OncoFAP uptake was reliably observed at primary breast cancer lesions, lymph node, and distant metastases as depicted in conventional imaging. In addition, ^68^Ga-OncoFAP-PET identified or substantiated suspicion for additional lesions, e.g., probable lymph node metastases at the internal mammary chain in two patients (Suppl. Fig. 8). Similarly, ^68^Ga-OncoFAP-PET supported clinical workup of non-breast-cancer patients, e.g., by depicting a peritoneal metastasis of colon cancer (SUV_max_ 20.0), supporting radiation therapy planning in non-FDG-avid fibrosarcoma (SUV_max_ 6.9), and identifying local relapse in post-transplant hepatocellular carcinoma (SUV_max_ 9.7) (Suppl. Fig. 9).

## Discussion

OncoFAP has been recently introduced as a new very high-affinity ligand with striking FAP targeting in small animals upon fluorophore- and ^177^Lu-labeling [15]. Here, we demonstrate the feasibility of ^68^Ga-radiolabeling and highly favorable targeting properties in small animal and clinical PET imaging in patients with cancer, validating ^68^Ga-OncoFAP as a new powerful alternative to clinically established PET tracers.

^68^Ga-labeled clinical PET tracers require a reliable radiosynthesis, high radiochemical yields, and high radiochemical purities based on an efficient, rapid, and simple labeling procedure using state-of-the-art equipment. ^68^Ga-OncoFAP-DOTAGA labeling fulfilled these requirements without reservation on a manual and a fully automated synthesis module. The determined low lipophilicity was advantageous for imaging application since the renal excretion route is favored and low background can be obtained. The high FAP binding affinity and metabolic stability were ideally suited for whole-body PET imaging justifying its exploration in small animals and clinical translation.

In vivo blocking experiments are traditionally employed to proof specific binding of PET tracers. We used a different approach of simultaneously implanting individual animals with a FAP-negative and a genetically altered FAP-expressing and otherwise identical tumor line. Accordingly, the observed manyfold difference of uptake between FAP^+^/FAP^−^ tumor lines can be solely attributed to specific tracer binding. We further substantiated this reasoning by applying pharmacokinetic modeling based on an extracorporeally derived arterial input function. Here, FAP^+^ and FAP^−^ tumors had similar results for modeling parameters associated with perfusion, permeability, and passive retention, whereas parameters reflecting specific target binding were significantly different.

The radiotracers FAPI-02, FAPI-04, and FAPI-46 developed at the University of Heidelberg, Germany, were the first available radiotracers with excellent imaging properties [4, 5]. Other FAP radiotracers appear to be so far used only in few centers as, e.g., [68Ga]Ga-DOTA.SA.FAPi [21, 22], or FAP-2286 [13]. In lack of direct comparison, it is currently unclear which of these radiotracers possess the optimal properties for PET imaging. To evaluate the competitiveness of OncoFAP, we compared it to the well-established tracer FAPI-46. OncoFAP was recently described as the small organic FAP ligand with the highest affinity [15]. Our observed higher inhibitory activity in a fluorescence-based FAP enzymatic assay for ^nat^Ga-OncoFAP compared to ^nat^Ga-FAPI-46 well-substantiates this assessment. We further benchmarked OncoFAP in head-to-head in vivo biodistribution studies against FAPI-46. Again, we observed higher ^68^Ga-OncoFAP uptake in murine FAP-positive tumors after 1 h in PET imaging and γ-counting. Also, the significant difference between the tracers in pharmacokinetic modeling for parameters k3/k4 and Vs confined to FAP^+^ tumors likely reflects a difference in FAP affinity. Washout characteristics of the two tracers, which are relevant for future theranostic applications, as assessed by the 3 h imaging time point, were not found to be statistically different.

Clinical imaging performance of ^68^Ga-OncoFAP is well in line with prior experience in our center using ^68^Ga-FAPI-46 [10]. In the group of 12 patients, the tracer reliably bound to primary cancers, lymph nodes, and distant metastases and is rapidly cleared from unaffected organs. Notably, contrasting the higher-than-expected hepatic uptake in preclinical ^68^Ga-OncoFAP biodistribution, the only significant difference in tracer uptake was a lower ^68^Ga-OncoFAP uptake in the liver compared to ^68^Ga-FAPI-46, rendering relevant hepatic tracer metabolism unlikely at least in humans. Mirroring our recently published results with ^68^Ga-FAPI-46 in breast cancer [10], ^68^Ga-OncoFAP-PET imaging led to establish or substantiate novel sites of disease and facilitated workup in a variety of clinical scenarios. Uptake in cancer was not significantly different between the tracers in our small study sample. Thus, future studies have to investigate whether the experimentally observed better affinity and higher uptake in tumor models finally translates to a superior contrast of ^68^Ga-OncoFAP in clinical cancer imaging.

Our study features limitations. The preclinical head-to-head comparison of ^68^Ga-OncoFAP with ^68^Ga-FAPI-46 is based on a small number of tumor bearing mice that in part redundantly contributed to data for gamma counting, PET quantification, and PK modeling, leading to remaining statistical uncertainties. This explains why we do not overemphasize this comparison. Analysis of clinical translation is retrospective and the number of patients is rather small. The clinical comparison of ^68^Ga-OncoFAP and ^68^Ga-FAPI-46 is underpowered and possibly features selection bias.

In conclusion, excellent preclinical and clinical PET imaging characteristics validate ^68^Ga-OncoFAP as a powerful alternative to currently available FAP tracers. Prospective studies are needed to define its accuracy in relevant clinical scenarios. Moreover, the potential of OncoFAP to deliver therapeutic payloads to cancer requires further preclinical and clinical investigation.

## Supplementary information

ESM 1 (DOCX 3.82 mb)

## Data Availability

Additional data is available in the supplement.

## Code availability (software application or custom code)

Does not apply
