# Supplementary material for: Translational imaging of the fibroblast activation protein (FAP) using the new ligand [68Ga]Ga-OncoFAP-DOTAGA
Source: Eur J Nucl Med Mol Imaging. 2021 Dec 27;49(6):1822–32. doi: 10.1007/s00259-021-05653-0 (PMC9016025; doi:10.1007/s00259-021-05653-0)
Supplement: Supplementary file 1 — (DOCX 3.82 mb) [file 259_2021_5653_MOESM1_ESM.docx]

# Materials and Methods

## Radiochemistry and -pharmacy. General Methods.

The radiosynthesis was carried out on a PET tracer radiosynthesizer (miniAllinOne (miniAiO), Trasis, Ans, Belgium) or a manual iQS Ga-68 Fluidic Labeling Module (itG, Garching, Germany). The recorded data were processed by the Trasis Supervision software (Trasis, Ans, Belgium) when the miniAiO was used. A pharmaceutical grade ^68^Ge/^68^Ga generator (GalliaPharm, Eckert & Ziegler Radiopharma GmbH, Berlin, Germany) with a calibration activity of 1.85 GBq was used for ^68^Ga-labelling. Quality control (QC) parameters were determined with the following devices and methods: Color, clearness and particle status of the batch solution were checked by visual inspection. The pH-value was measured with a pH 1000 L pH meter (VWR International GmbH, Darmstadt, Germany). Radiochemical purity and identity via thin layer chromatography (TLC) were tested with an iTLC-SG paper strip (20 mm x 150 mm, 100 mm migration distance, Agilent Technologies, Santa Clara, US) using 0.5 M NH_4_OAc/MeOH 1/1 (v/v) as mobile phase. The TLC strip was analyzed with a MiniGita radio-TLC scanner and the corresponding GINA Star software (Elysia-Raytest GmbH, Straubenhardt, Germany). Radiochemical purity and identity via high performance liquid chromatography (HPLC) were determined using an analytical gradient radio-HPLC system **A** composed of a P6.1L (APH35EA) pump and a UVD 2.1L (ADA01XA) UV detector (Knauer Wissenschaftliche Geräte GmbH, Berlin, Germany), a GabiStar γ-detector (Elysia-Raytest GmbH, Straubenhardt, Germany) and a Eurospher II 100-5 C18 column (150 mm × 4 mm, Knauer Wissenschaftliche Geräte GmbH, Berlin, Germany). HPLC method **A** started with a linear gradient from 0 % to 30 % CH_3_CN in water (0.1 % TFA) over 9 min, holding for 3 min followed by a linear gradient from 30 % to 0 % CH_3_CN in water (0.1 % TFA) over 2 min, with λ=214 nm and a flow rate of 2.0 mL·min^-1^. The recorded data of the HPLC-system were processed by the GINA Star software (Elysia-Raytest GmbH, Straubenhardt, Germany). Ethanol concentration of the batch solution was measured with gas chromatography (GC) using a 7890A gas chromatograph (Agilent Technologies, Santa Clara, US) with a polar deactivated guard column (5 m, ID 0.32 mm, Restek GmbH, Bad Homburg, Germany) and a Rtx 200 column (30 m, ID 0.32 mm, Restek GmbH, Bad Homburg, Germany). The data analysis was also performed by the GINA Star software (Elysia-Raytest GmbH, Straubenhardt, Germany). Bacterial endotoxins were determined with an Endosafe-Nexgen portable test system (PTS) device (Charles River Laboratories, Wilmington, US) using disposable test cartridges that contain synthetic color-producing substrate. The quantitative detection of endotoxins in this test system is based on kinetic chromogenic methods. Filter integrity testing was performed within the miniAiO synthesizer using a hydrophilic non-vented 0.22 µm PharmAssure (HP1002) sterile filter (Pall corporation, New York, US) with a bubble-point of ≥ 3172 mbar. Identity of the radionuclide was verified by the measurement of the half-life with an isomed 2010 activimeter (Nuvia Instruments GmbH, Dresden, Germany). Radionuclide purity was determined by the measurement of the γ-spectrum of the batch at several time points using an isomed 2100 γ-spectrometer (Nuvia Instruments GmbH, Dresden, Germany). Sterility testing was performed by the Institute of Hygiene, University Hospital Münster (Germany) in accordance with the Ph. Eur. standards (Ph. Eur. 10.0/2.06.01.00).

## Synthesis of [^nat^Ga]GaOncoFAP-DOTAGA.

OncoFAP-DOTAGA (20 mg, 0.021 mmol, 1 eq.) was dissolved in acetate buffer, pH=4.5 (1.8 mL). Subsequently a solution of GaCl_3_ (37 mg, 0.21 mmol, 10 eq.) dissolved in 1 N HCl (0.2 mL) was added. The reaction was stirred at 90°C for 10 min, then cooled down to room temperature. and purified via RP-HPLC (Agilent 1200 series system equipped with Synergi 4μm Polar-RP 80Å 10 × 150 mm C18 column using a gradient of 90:10 to 50:50 water/acetonitrile + 0.1% TFA in 7 min). The desired fractions were collected and lyophilized to afford a pale-yellow solid (13 mg, 62%).

[M+H]^+^= 1026.2 (^69^Ga), 1028.2 (^71^Ga).


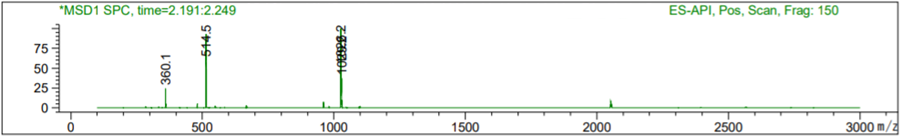


^1^H NMR (500 MHz, DMSO-*d*_6_) δ 10.15 (s, 1H), 9.16 (t, *J=*6.0 Hz, 1H), 9.02 (d, *J=*4.4 Hz, 1H), 8.66 (d, *J=*7.7 Hz, 1H), 8.00 (d, *J=*8.5 Hz, 1H), 7.97 (t, *J=*5.7 Hz, 1H), 7.92 – 7.85 (m, 1H), 7.68 – 7.62 (m, 2H), 5.19 (d, *J=*9.1 Hz, 1H), 4.38 – 4.29 (m, 1H), 4.25 (t, *J=*6.1 Hz, 1H), 4.22 – 4.06 (m, 2H), 3.86 – 3.72 (m, 4H), 3.72 – 3.52 (m, 6H), 3.52 – 3.39 (m, 2H), 3.39 – 3.18 (m, 4H), 3.17 – 2.98 (m, 4H), 2.98 – 2.88 (m, 2H), 2.85 – 2.80 (m, 3H), 2.59 – 2.44 (m, 8H), 2.43 – 2.34 (m, 3H), 1.97 – 1.80 (m, 2H).

^13^C NMR (126 MHz, DMSO-*d*_6_) δ 173.03, 172.26, 171.95, 171.61, 170.38, 169.92, 169.64, 168.26, 167.62, 148.94, 143.07, 138.70, 135.23, 128.13, 124.68, 120.17, 119.81, 118.31, 117.22, 60.75, 60.59, 60.52, 60.04, 57.50, 56.29, 55.46, 55.12, 54.44, 53.76, 51.68 (t, *J*=31.5 Hz), 50.17, 44.74, 44.69, 41.82, 39.11, 38.62, 36.87 (t, *J*=24.6 Hz), 36.3, 32.8, 32.63, 30.96, 20.44.


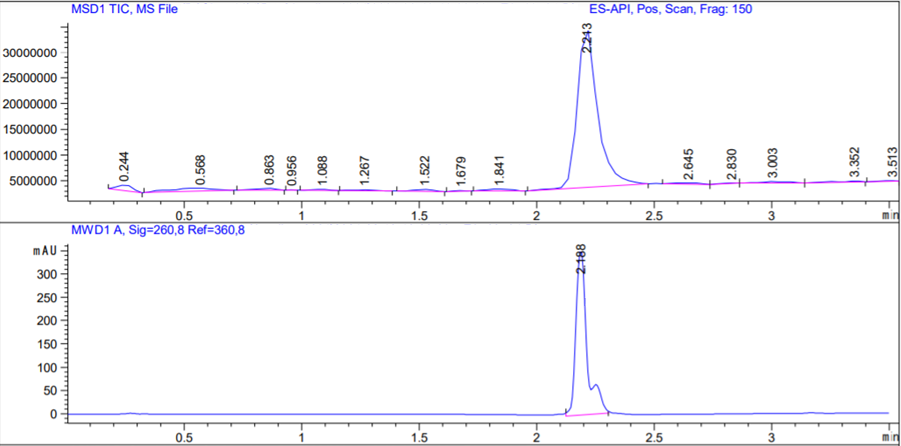


## Synthesis of [^nat^Ga]FAPI-46.

FAPI-46 (20 mg, 0.023 mmol, 1 eq.) was dissolved in acetate buffer, pH=4.5 (1.8 mL). Subsequently a solution of GaCl_3_ (40 mg, 0.23 mmol, 10 eq.) dissolved in 1 N HCl (0.2 mL) was added. The reaction was stirred at 90°C for 10 min, then cooled down to room temperature and purified via RP-HPLC (Agilent 1200 series system equipped with Synergi 4μm Polar-RP 80Å 10 × 150 mm C18 column using a gradient of 90:10 to 50:50 water/acetonitrile + 0.1% TFA in 7 min). The desired fractions were collected and lyophilized to afford a pale-yellow solid (11 mg, 50%).

[M+H]^+^= 951.6 (^69^Ga), 953.3 (^71^Ga).


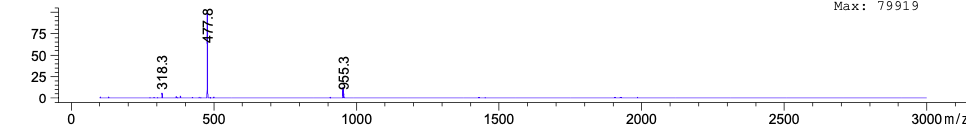


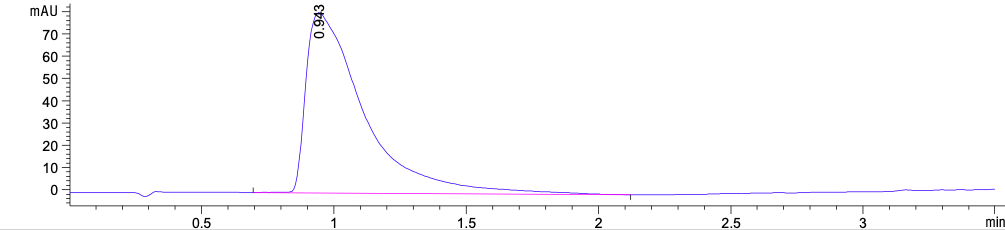


## Radiosynthesis of [^68^Ga]GaOncoFAP-DOTAGA on the Trasis miniAllinOne (miniAIO) module

2,2',2''-(10-(1-carboxy-4-((2-(4-((4-((2-((S)-2-cyano-4,4-difluoropyrrolidin-1-yl)-2-oxoethyl)carbamoyl)quinolin-8-yl)amino)-4-oxobutanamido)ethyl)amino)-4-oxobutyl)-1,4,7,10-tetraazacyclododecane-1,4,7-triyl)triacetic acid (Onco-FAP-DOTAGA) was synthesized as shown in **Scheme 1** [1] and stored at -20°C in 0.625 M sodium acetate buffer solutions (with ascorbic acid as a stabilizer [2]). Precursor solutions (concentration: 20 µg/mL) in portions of 24 µg (≡ 25 nmol OncoFAP-DOTAGA per portion dissolved in 1200 µL buffer solution (pH ~ 6.5)), prefilled in single use vials, compatible with the disposable cassette were used. One exemplary vial, prefilled in the described way, was tested for sterility before use. Radiolabeling was performed on the legal basis of German Pharmaceuticals Act (AMG §13(2b), i. e. magistral preparation) according to **Scheme 1**. [^68^Ga]GaCl_3_ (T_½_=68 min, β^+^=89 % and EC=11 %) was obtained from a 50 mCi (1.85 GBq) ^68^Ge/^68^Ga radionuclide generator (EZAG, Berlin Germany, approval number 89872.00.00) and the eluate was used without pre-purification. A disposable cassette-based fully automated labeling module (miniAllinOne (miniAIO), Trasis, Ans, Belgium) was used for the radiolabeling procedure. The regarding single-use cassette was purchased from Trasis (Ans, Belgium). All reagents (except for the precursor solution) were taken from a disposable reagent kit (GMP quality, Trasis, Ans, Belgium) suitable for the ^68^Ga-labeling of peptides and the procedure, recommended by the manufacturer [3] was used. The following items were mounted on the cassette prior to synthesis: one prefilled vial containing the precursor solution, one prefilled vial containing 5 mL ethanol, one plastic bag with 50 mL isotonic saline and one syringe containing 5 mL 0.1 HCl (0.36 %). The synthesis was started. Prior to elution of the generator, the content of the precursor vial was transferred to the reactor. The ^68^Ga radioisotope (acc. to Ph. Eur. monograph 2464) was eluted (flow: 2 mL/min) from the ^68^Ge/^68^Ga generator with the contents of the HCl syringe and directly transferred into the reaction vessel without pre-purification. After incubation for 7 min at 120-110°C the reaction mixture was diluted with 9 mL of isotonic NaCl (B. Braun, Melsungen Germany) and loaded onto a preconditioned Oasis HLB SPE cartridge (Waters, Eschborn, Germany), where the formed [^68^Ga]Ga-OncoFAP-DOTAGA was trapped. The reactor was rinsed with 5 mL 0.9 % saline and the rinse solution was used to wash the SPE cartridge. The SPE cartridge was washed with additional 3 mL isotonic saline and eluted from the SPE cartridge in the product vial using 0.5 mL ethanol. The elution solution was passed through a 0.22 µm sterile filter and formulated with 9.5 mL of isotonic saline, used for the final rinsing of the cartridge. Aliquots were sampled for QC, sterility testing, endotoxin test and as a reference sample. The quality control of the final product solution, obtained by the described procedure was performed by HPLC, TLC, GC, endotoxin- and sterility testing, spectrometry, integrity testing of the sterile filter as well as determination of pH and half-life. Filter integrity was tested online by a built-in device following synthesis using the bubble point method. The identity of the product was confirmed by comparing the HPLC retention times of the product and its non-radioactive analog, synthesized as pointed out in **Suppl. Figure 2**. By following the described procedure [^68^Ga]Ga-OncoFAP-DOTAGA was synthesized within 19 min overall synthesis time. The radiochemical purity (rcp) of the resulting injection solution was 98.1 ± 1.1 % (HPLC, n=16) and the radiochemical yield (rcy) was 75.3 ± 2.9 % (n=16, not corrected for decay) at the EOS. All QC parameters (see **Suppl. Table 1**) were determined in accordance with the Ph. Eur. standards given for ^68^Ga‑DOTA-TOC (monograph 2482).

## Radiosynthesis of [^68^Ga]Ga-OncoFAP-DOTAGA on the ITG iQS Ga-68 Fluidic Labeling module

2,2',2''-(10-(1-carboxy-4-((2-(4-((4-((2-((S)-2-cyano-4,4-difluoropyrrolidin-1-yl)-2-oxoethyl)carbamoyl)quinolin8-yl)amino)-4-oxobutanamido)ethyl)amino)-4-oxobutyl)-1,4,7,10-tetraazacyclododecane-1,4,7-triyl)triacetic acid (Onco-FAP-DOTAGA) was synthesized as shown in **Scheme 1** [1] and stored in 0.625 M sodium acetate buffer solutions (with ascorbic acid as a stabilizer [2]). Precursor solutions (concentration: 20 µg/mL) in portions of 24 µg (≡ 25 nmol OncoFAP-DOTAGA per portion dissolved in 1200 µL buffer solution (pH ~ 6.5)), prefilled in single use vials, compatible with the disposable cassette were used. [^68^Ga]GaCl_3_ (T½=68 min, β^+^=89 % and EC=11 %) was obtained from a 50 mCi (1.85 GBq) ^68^Ge/^68^Ga radionuclide generator (EZAG, Berlin Germany, approval number 89872.00.00) and the eluate used without pre-purification. A disposable-cassette-based manual iQS Ga-68 Fluidic Labeling Module (itG, Garching, Germany) was used for the radiolabeling procedure. All reagents were taken from a disposable reagent kit (GMP quality, itG, Garching, Germany) for the ^68^Ga-labeling of peptides and the procedure, recommended by the manufacturer [4] was used. The ^68^Ga radioisotope was manually eluted (flow: approx. 2.0‑2.5 mL/min) from the ^68^Ge/^68^Ga generator using hydrochloric acid (HCl, 5.0 mL, 0.1 M) and directly transferred into the reaction vessel, containing a pre-heated (~100°C) mixture of 1.2 mL OncoFAP-DOTAGA solution (prepared as described above), buffered with 0.625 M sodium acetate buffer and ascorbic acid as a stabilizer. After incubation for 10 min at ~100°C the reaction mixture was loaded onto a preconditioned C18 Light SPE cartridge. The reactor was rinsed with 5.0 mL 0.9 % NaCl and the rinse solution was also loaded on the C18 cartridge. [^68^Ga]Ga-OncoFAP-DOTAGA was eluted from the C18 cartridge in the product vial using 1.1 mL of an ethanol/water mixture (3:2), passing through a 0.22 μm sterile filter and formulated with 5.0 mL of 0.9% NaCl, used for the final rinsing of the cartridge. The quality control of the final product solution, obtained by the described procedure was performed by HPLC, TLC, GC, endotoxin- and sterility testing, gamma spectrometry, integrity testing of the sterile filter as well as determination of pH and half-life. Filter integrity was tested online by an external device following synthesis using the bubble point method. The identity of the product was confirmed by comparing the HPLC retention times of the product and its non-radioactive analog, synthesized as pointed out in **Suppl. Figure 2**. [^68^Ga]Ga-OncoFAP-DOTAGA was synthesized within 25-30 min synthesis time. The radiochemical purity was 97.5 ± 1.1 % (HPLC, n=16) and the radiochemical yield was 69.1 ± 12.7 % (n=16). As the module offers no device for measuring the start activity, the theoretical activity after elution of the radionuclide generator was calculated according to the SmPC of the manufacturer. All QC parameters were in accordance with the Ph. Eur. standards given for ^68^Ga-DOTA-TOC (monograph 2482).

## *In vitro* stability of [^68^Ga]Ga-OncoFAP-DOTAGA in human and mouse serum.

The stability of [^68^Ga]Ga-OncoFAP-DOTAGA in human and murine serum was measured after incubation at 37°C for 120 min. Therefore, an aliquot of formulated solution (mouse: 30 µL, ~6.3-6,6 MBq; human: 50 µL, ~5 MBq and 100 µL, ~10 MBq) was added to a tube with blood serum (200 µL) and the mixture was shaken at 37°C. After 10, 30, 60, 90, 120 min an aliquot (20 µL) of the respective radioligand was taken out of the tube and added to cooled ACN (100 µL). The sample was analyzed by analytical radio-HPLC (method **A**) after centrifugation for 5 min.

## Determination of the log*D*7.4–value of [^68^Ga]Ga-OncoFAP-DOTAGA.

The lipophilicity of [^68^Ga]Ga-OncoFAP-DOTAGA was determined similar to the procedure described by Prante *et* al. [5]. An aliquot of the radioligand (~ 980 kBq) in PBS buffer (10 μL, pH 7.4) was added to PBS buffer (590 μL, pH 7.4) and octan-1-ol (600 μL). The two-layer mixture was shaken for 10 min on a vortex mixer at RT and centrifuged (3000 rpm) for 5 min. Three samples were prepared and 100 μL of both layers were measured in a γ-counter 2480 Wizard2 (Perkin-Elmer, Waltham, USA). The partition coefficient was determined by dividing cpm (octanol) by cpm (PBS) and indicated as log*D*7.4.

## *In vitro* inhibition assay of prolyl peptidases

The synthetic fluorometric DPP substrate 1 (BPS Bioscience, San Diego, CA, USA) Catalog no. 80305) was used to assay Dipeptidyl peptidase-8 (DPP-8, BPS Bioscience, Catalog no. 80080), Prolyl oligopeptidase (POP, BPS Bioscience, Catalog no. 80105), and Fibroblast Activation Protein (FAP, BPS Bioscience, Catalog no. 80100) following the instructions in the protocols of the assay kit supplier. The inhibition was assayed by preincubating DPP-8 human recombinant enzyme (20 ng/reaction for DDP‑8), 200 ng/reaction for POP, and 250 ng/reaction for FAP) and inhibitor compounds Talabostat, S 17092, [^nat^Ga]FAPI-46 and [^nat^Ga]Ga-OncoFAP-DOTAGA at varying concentrations (10 pM–100 µM) in DPP Assay buffer (BPS Bioscience, 80300) at 37°C for 10 min. An aliquot of substrate (5 mL of a 100 mM solution for DPP-8 and FAP, 5 mL of a 50 mM solution for POP) was then added to 95 mL of the preincubated prolyl peptidase/inhibitor mixture, and the fluorescence was determined at 37°C by following product release over time. The changes in fluorescence were monitored using a Microplate Analyzer (Tristar2 Multimode Reader LB 942, Berthold Technologies, Bad Wildbad, Germany) with excitation and emission wavelengths set to 360 and 460 nm, respectively. Reaction rates were measured from the initial 30 min of the reaction profile where product release was linear with time and plotted as a function of inhibitor dose. From the resulting inhibition curves, the IC_50_ values for each inhibitor were calculated by non-linear regression analysis, performed using the Grace 5.1.25 software (Linux).

## References

1. Millul J, Bassi G, Mock J, Elsayed A, Pellegrino C, Zana A, et al. An ultra-high-affinity small organic ligand of fibroblast activation protein for tumor-targeting applications. Proc National Acad Sci. 2021;118:e2101852118.

2. Mu L, Hesselmann R, Oezdemir U, Bertschi L, Blanc A, Dragic M, et al. Identification, characterization and suppression of side-products formed during the synthesis of high dose ^68^Ga-DOTA-TATE. Appl Radiat Isotopes Incl Data Instrum Methods Agric Industry Medicine. 2013;76:63–9.

3. AllinOne and miniAllinOne Specific Application Manual, [68Ga]Ga-Peptide, Without pre-purification, Trasis, Ans, Belgium.

4. itG Ga-68 Radiolabeling KIT, Operating Manual, Version 1.0, Status 01/2014, itG, Garching, Germany, 2014.

5. Prante O, Hocke C, Löber S, Hübner H, Gmeiner P, Kuwert T. Tissue distribution of radioiodinated FAUC113: assessment of a pyrazolo(1,5-a) pyridine based dopamine D4 receptor radioligand candidate. Nuklearmedizin. 2006;45:41–8.

# Supplementary Tables

| **test/QC parameter** | **result** | **acceptance criterium** | **corresponds (yes/no)** |
| --- | --- | --- | --- |
| appearance | corresponds | clear, colorless, free of visible particles | yes |
| pH | 4.61 ± 0.17 (n=13) | 4.0 < pH < 8.0 | yes |
| batch radioactivity (per V_max_=10 mL) | 1208-636 MBq (n=8)  (depending on number of elutions performed with generator used) | n. a. |  |
| molar activity | 25.4-48.3 MBq/nmol | n. a. |  |
| activity concentration | 63.6-120.8 MBq/mL (n=8)  (depending on number of elutions performed with generator used) | n. a. |  |
| radiochemical purity (HPLC) | 98.1 ± 1.1 % (n=16) | ≥ 92 % | yes |
| radiochemical purity (TLC) | ≥ 99 % (n=13) | ≥ 92 % | yes |
| radiochemical identity (HPLC) | R_t_ [^68^Ga]Ga-OncoFAP-DOTAGA:  467 ± 17 s (n=11)  R_t_ reference standard: 466 ± 16 s (n=11) | R_t_ [^68^Ga]Ga-OncoFAP-DOTAGA  corresponds to R_t_ reference standard | yes |
| relative standard deviation (HPLC Peak [^68^Ga]Ga-OncoFAP-DOTAGA and reference standard) | 0.71-7.78 % (n=11) | n. a. |  |
| radiochemical identity (TLC) | R_f_ [^68^Ga]Ga-OncoFAP-DOTAGA: 0.86 ± 0.06 (n=13) | R_f_ [^68^Ga]Ga-OncoFAP-DOTAGA: 0.7-1.0 | yes |
| ethanol (GC) | 54.6 ± 19.1 mg/mL (n=13) | ≤ 2500 mg/day | yes |
| endotoxins | < 2.5 EU/mL | ≤ 17.5 EU/mL | yes |
| radionuclidic purity | ≥ 99.9 % ^68^Ga and  -Energie=511 keV | corresponds | yes |
| radionuclidic identity | T_1/2=_67.44 ± 0.65 min | T_1/2=_62-74 min | yes |
| sterility | sterile | sterile | yes |

***Suppl. Table 1.*** *Product specification for [^68^Ga]Ga-OncoFAP-DOTAGA. All syntheses were performed on the Trasis miniAIO module. An amount of 25 nmol precursor was used for each synthesis. Tests performed or finished after product release are highlighted in grey. Test with no acceptance criterium (n. a.) are not part of the UKM-specific release specification. For HPLC conditions (method* ***A****) see section Radiochemistry and -pharmacy. General Methods.*

| **organ / material** | **mean ± std OncoFAP 1 h p.i. [% ID/g] (n=6)** | **mean ± std FAPI-46 1 h p.i. [% ID/g] (n=4)** | **p-value** | **mean ± std organ-to-blood ratio OncoFAP** | **mean ± std organ-to-blood ratio FAPI-46** |
| --- | --- | --- | --- | --- | --- |
| **urine** | 426.18 ± 206.71 | 525.55 ± 279.13 | .61 |  |  |
| **blood** | 0.37 ± 0.24 | 0.69 ± 0.15 | .07 |  |  |
| **plasma** | 0.54 ± 0.41 | 1.02 ± 0.30 | .07 |  |  |
| **brain** | 0.03 ± 0.41 | 0.04 ± 6.99 | .26 | 0.09 ± 0.03 | 0.06 ± 0.01 |
| **heart** | 0.15 ± 0.08 | 0.25 ± 0.07 | .11 | 0.41 ± 0.04 | 0.36 ± 0.04 |
| **kidney** | 2.35 ± 2.25 | 8.58 ± 7.52 | .04 | 5.67 ± 1.60 | 11.58 ± 8.84 |
| **liver** | 0.40 ± 0.16 | 0.46 ± 0.06 | .35 | 1.20 ± 0.47 | 0.70 ± 0.23 |
| **lung** | 0.49 ± 0.23 | 1.17 ± 0.43 | .04 | 1.38 ± 0.25 | 1.84 ± 1.04 |
| **muscle** | 0.14 ± 0.07 | 0.21 ± 0.07 | .26 | 0.41 ± 0.07 | 0.31 ± 0.11 |
| **spleen** | 0.27 ± 0.11 | 0.29 ± 0.05 | .76 | 0.80 ± 0.29 | 0.43 ± 0.06 |
| **tumor FAP-** | 0.31 ± 0.13 | 0.38 ± 0.12 | .47 | 0.9 ± 0.19 | 0.55 ± 0.08 |
| **tumor FAP+** | 2.49 ± 0.56 | 1.28 ± 0.40 | **.01*** | 8.61 ± 5.1 | 1.98 ± 0.92 |

***Suppl. Table 2****. Gamma counting results as head-to-head comparison between [^68^Ga]Ga-OncoFAP-DOTAGA and [^68^Ga]Ga-FAPI-46 1 h p.i. Only accumulation of FAP+ tumors was significantly different between the two tracers after correcting for multiple comparisons. For comparison of 10 organs / materials, a Bonferroni correction leads to a corrected p-value threshold for significance of p<.005. For the 2 tumor types, Bonferroni correction leads to a corrected p-value threshold of p<.025. * indicates significance.*

| **organ / material** | **mean ± std OncoFAP 3 h p.i. [% ID/g] (n=6)** | **mean ± std FAPI-46 3 h p.i. [% ID/g] (n=6)** | **p-value** | **mean ± std organ-to-blood ratio OncoFAP** | **mean ± std organ-to-blood ratio FAPI-46** |
| --- | --- | --- | --- | --- | --- |
| **blood** | 0.08 ± 0.03 | 0.22 ± 0.31 | .48 |  |  |
| **plasma** | 0.13 ± 0.05 | 0.38 ± 0.53 | .24 |  |  |
| **brain** | 0.01 ± 0.00 | 0.01 ± 0.00 | .94 | 0.16 ± 0.04 | 0.15 ± 0.10 |
| **heart** | 0.04 ± 0.01 | 0.05 ± 0.02 | .59 | 0.61 ± 0.12 | 0.51 ± 0.23 |
| **kidney** | 0.65 ± 0.21 | 0.98 ± 0.60 | .13 | 9.55 ± 4.92 | 10.39 ± 5.81 |
| **liver** | 0.76 ± 0.11 | 0.14 ± 0.03 | **.002*** | 10.9 ± 2.87 | 1.73 ± 1.28 |
| **lung** | 0.20 ± 0.04 | 0.17 ± 0.06 | .31 | 2.95 ± 1.10 | 2.27 ± 1.98 |
| **muscle** | 0.04 ± 0.03 | 0.04 ± 0.02 | .94 | 0.54 ± 0.17 | 0.38 ± 0.26 |
| **spleen** | 0.20 ± 0.08 | 0.07 ± 0.02 | **.002*** | 2.30 ± 1.60 | 0.78 ± 0.47 |
| **tumor FAP-** | 0.10 ± 0.03 | 0.11 ± 0.03 | .99 | 1.44 ± 0.44 | 1.15 ± 0.67 |
| **tumor FAP+** | 2.60 ± 1.96 | 2.64 ± 0.60 | .59 | 38.06 ± 33.08 | 28.62 ±17.69 |

***Suppl. Table 3****. Gamma counting results as head-to-head comparison between [^68^Ga]Ga-OncoFAP-DOTAGA and [^68^Ga]Ga-FAPI-46 3 h p.i. Urine could not be reliably collected 3 h p.i. Only uptake in liver and spleen was significantly different between the two tracers after correcting for multiple comparisons. For comparison of 9 organs / materials, a Bonferroni correction leads to a corrected p-value threshold for significance of p<.006. For the 2 tumor types, Bonferroni correction leads to a corrected p-value threshold of p<.025. * indicates significance.*

| ***organ / material*** | ***p-value 1 h vs 3 h OncoFAP (n=6 vs 6)*** | ***p-value 1 h vs 3 h FAPI-46 (n=4 vs 6)*** |
| --- | --- | --- |
| **blood** | **.002*** | .07 |
| **plasma** | .03 | .11 |
| **brain** | **.002*** | .01 |
| **heart** | **.002*** | .01 |
| **kidney** | .009 | .01 |
| **liver** | **.002*** | .01 |
| **lung** | **.004*** | .01 |
| **muscle** | .009 | .01 |
| **spleen** | .31 | .01 |
| **tumor FAP-** | **.002*** | **.01*** |
| **tumor FAP+** | .67 | **.01*** |

***Suppl. Table 4.*** *Comparison of gamma counting results of different time points for the same tracer 1 h and 3 h p.i.. For comparison of 9 organs / materials, a Bonferroni correction leads to a corrected p-value threshold for significance of p<.006. For the 2 tumor types, Bonferroni correction leads to a corrected p-value threshold of p<.025. * indicates significance.*

| ***organ*** | ***OncoFAP 10 min  [SUV_mean_] (n=11)*** | ***FAPI-46 10 min [SUV_mean_] (n=10)*** | ***p-value OncoFAP vs FAPI-46*** | ***OncoFAP 1 h [SUV_mean_] (n=11)*** | ***FAPI-46 1 h [SUV_mean_] (n=10)*** | ***p-value OncoFAP vs FAPI-46*** | ***OncoFAP 3 h [SUV_mean_] n=5*** | ***FAPI-46 3 h [SUV_mean_] n=6*** | ***p-value OncoFAP vs FAPI-46*** |
| --- | --- | --- | --- | --- | --- | --- | --- | --- | --- |
| ***blood*** | 0.49 ± 0.06 | 0.52 ± 0.09 | *.55* | 0.20 ± 0.08 | 0.19 ± 0.07 | *>.99* |  |  |  |
| ***kidney*** | 1.18 ± 0.65 | 1.43 ± 0.88 | *.64* | 0.25 ± 0.12 | 0.54 ± 0.54 | *.13* | 0.07 ± 0.01 | 0.07 ± 0.02 | *.66* |
| ***liver*** | 0.45 ± 0.10 | 0.57 ± 0.17 | *.04* | 0.14 ± 0.04 | 0.28 ± 0.16 | ***.002**** | 0.06 ± 0.04 | 0.28 ± 0.19 | *.02* |
| ***muscle*** | 0.32 ± 0.06 | 0.35 ± 0.07 | *.42* | 0.07 ± 0.03 | 0.08 ± 0.04 | *.86* | 0.02 ± 0.00 | 0.02 ± 0.00 | *>.99* |
| ***spleen*** | 0.34 ± 0.08 | 0.40 ± 0.19 | *>.99* | 0.10 ± 0.02 | 0.15 ± 0.07 | *.053* | 0.03 ± 0.01 | 0.09 ± 0.06 | *.08* |
| **tumor FAP-** | 0.35 ± 0.07 | 0.39 ± 0.07 | *.21* | 0.08 ± 0.04 | 0.10 ± 0.05 | *.42* | 0.02 ± 0.01 | 0.02 ± 0.01 | *.93* |
| **tumor FAP+** | 0.45 ± 0.08 | 0.40 ± 0.07 | *.25* | 0.38 ± 0.08 | 0.25 ± 0.06 | ***.004**** | 0.21± 0.06 | 0.16 ± 0.06 | *.25* |

***Suppl. Table 5.*** *SUV_mean_ of small animal PET dynamic measurements as head-to-head comparison between [^68^Ga]Ga-OncoFAP-DOTAGA and [^68^Ga]Ga-FAPI-46. Only uptake in the liver and FAP+ tumors 1 h p.i. was significantly different after correcting for multiple testing. Interestingly, stronger uptake in the liver for [^68^Ga]Ga-FAPI-46 contrasted the significantly higher gamma counting activity in the liver in [^68^Ga]Ga-OncoFAP-DOTAGA in a different set of mice (Suppl. Table 3). For comparison of 5 organs at 3 different time points Bonferroni correction leads to a corrected p-value threshold for significance of p<.003. For the 2 tumor types at 3 different time points, Bonferroni correction leads to a corrected p-value threshold of p<.008. * indicates significance.*

| ***organ*** | ***p-value 10 min vs 1 h OncoFAP (n=11 vs 11)*** | ***p-value 10 min vs 1 h FAPI-46 (n=10 vs 10)*** | ***p-value 1 h vs 3 h OncoFAP (n=11 vs 5)*** | ***p-value 1 h vs 3 h FAPI-46 (n=10 vs 6)*** |
| --- | --- | --- | --- | --- |
| ***blood*** | ***<.001**** | ***<.001**** | *-* | *-* |
| ***kidney*** | ***<.001**** | *.006* | ***<.001**** | ***<.001**** |
| ***liver*** | ***<.001**** | *.006* | *0.009* | *.71* |
| ***muscle*** | ***<.001**** | ***<.001**** | ***<.001**** | ***<.001**** |
| ***spleen*** | ***<.001**** | ***<.001**** | ***<.001**** | *.04* |
| **tumor FAP-** | ***<.001**** | ***<.001**** | ***<.001**** | ***<.001**** |
| **tumor FAP+** | 0.09 | ***<.001**** | 0.055 | ***.01**** |

***Suppl. Table 6.*** *Continuation of Suppl. Table 6. Comparison of different time points for the same tracer in dynamic PET measurements. For overall 9 comparisons of 5 organs at 3 time points, a Bonferroni correction leads to a corrected p-value threshold for significance of p<.006. For overall 4 comparisons of the 2 tumor types at 3 different time points, Bonferroni correction leads to a corrected p-value threshold of p<.013. * indicates significance.*

|  | ***OncoFAP tumor FAP- (n=6)*** | ***FAPI-46 tumor FAP- (n=4)*** | ***p-value OncoFAP vs FAPI-46*** | ***OncoFAP tumor FAP+ (n=6)*** | ***FAPI-46 tumor FAP+ (n=4)*** | ***p-value OncoFAP vs FAPI-46*** |
| --- | --- | --- | --- | --- | --- | --- |
| ***k1*** | *0.049* ± 0.010 | *0.049* ± 0.003 |  | *0.040* ± 0.009 | *0.042* ± 0.012 |  |
| ***k2*** | *0.331* ± 0.046 | *0.323*  ± 0.115 |  | *0.235* ± 0.034 | *0.298* ± 0.038 |  |
| ***k3*** | *0.015* ± 0.023 | *0.012* ± 0.031 |  | *0.062* ± 0.015 | *0.029* ± 0.024 |  |
| ***k4*** | *0.02* ± 0.032 | *0.015* ± 0.038 |  | *0.008* ± 0.004 | *0.017* ± 0.011 |  |
| ***k3/k4*** | *0.740* ± 0.825 | *0.801* ± 0.908 | *.76* | *7.913* ± 4.825 | *1.770* ± 2.737 | ***.04**** |
| ***Vs*** | *0.109* ± 0.14 | *0.121* ± 0.227 | *.91* | *1.336* ± 0.778 | *0.246* ± 0.287 | ***.02**** |
| ***Patlak slope*** | *0.000* ± 0.000 | *0.000* ± 0.000 | *.61* | *0.007* ± 0.005 | *0.002* ± 0.001 | ***.02**** |
| ***Patlak  intercept*** | *0.216* ± 0.061 | *0.207* ± 0.019 | *>.99* | *0.196* ± 0.074 | *0.176* ± 0.035 | *.61* |

***Suppl. Table 7.*** *Head-to-head comparison of [^68^Ga]Ga-OncoFAP-DOTAGA and [^68^Ga]Ga-FAPI-46 results of pharmacokinetic modeling of dynamic small animal PET using invasive measurements of the arterial input function (AIF) with an extracorporeal circulation and the Twilite measurement unit. 2-Tissue Compartment Model (2TCM) (k1-k4) and Patlak modeling were applied. Additionally ratio k3/k4 and distribution volume Vs are calculated for 2TCM. Values are displayed as geometric mean ± SD. k1 and k2 demonstrated only small differences between the two tracers in FAP- and FAP+ tumors, reflecting indifferent passive wash in and flush out of the tracers. In contrast k3, k4, demonstrated stronger differences and k3/k4 and Vs were heavily and significantly different between the two tracers only for FAP+ tumors, but not for FAP-. This finding strongly points towards a difference of affinity of the two tracers, rather than different passive properties. Consistently Patlak model demonstrated unchanged Patlak Intercept, and heavily and significantly different Patlak slope between the two tracers only for FAP+ tumors. No correction of multiple testing was performed, because of the strong interdependence of the tested variables. * indicates significance p<.05.*

# Supplementary Figures

***Suppl. Figure 1:*** *Commercially available FAP inhibitors Talabostat and S 17092 used as reference compounds in in-vitro inhibition assays of prolyl peptidases.*

***Suppl. Figure 2:*** *Synthesis of the non-radioactive reference compound [^nat^Ga]Ga-OncoFAP-DOTAGA: a. GaCl_3_, acetate buffer, 90°C, 62%.*

***
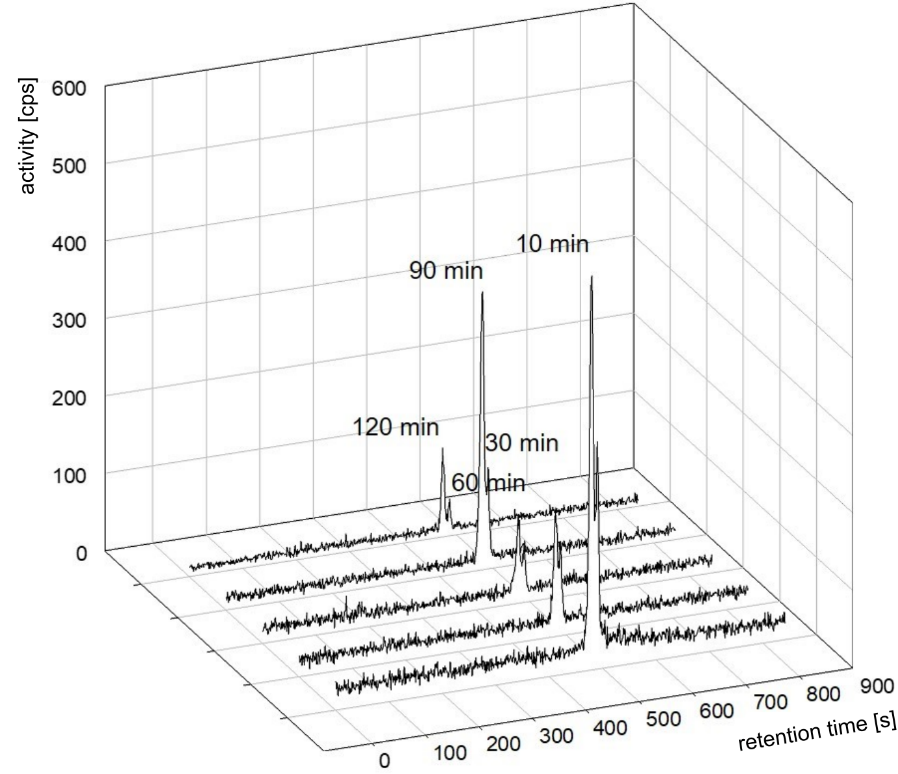
***

***Suppl. Figure 3****. Stability of [^68^Ga]Ga-OncoFAP-DOTAGA in mouse blood serum at 37 °C as analyzed by analytical radio-HPLC. Radio-HPLC chromatograms are shown for incubation times from 10 to 120 min.*

**
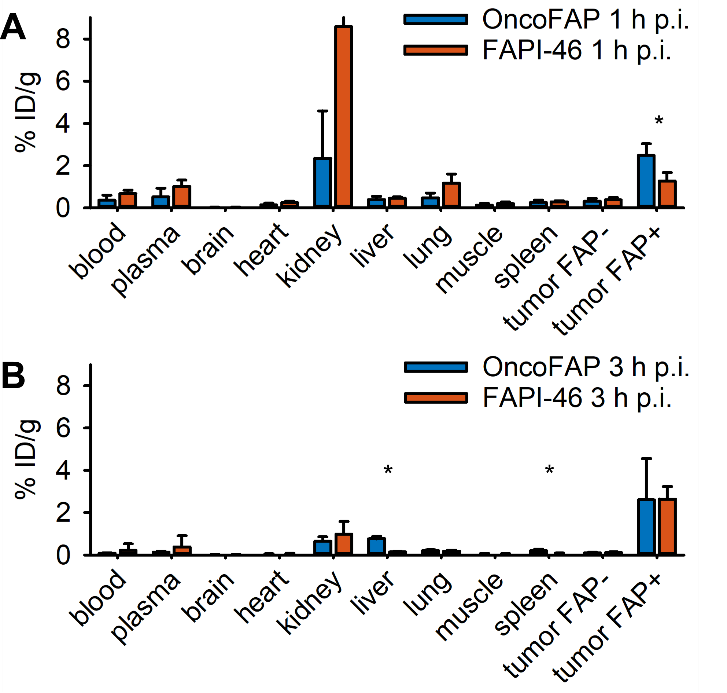
**

***Suppl. Fig. 4.*** *Head-to-head comparison of gamma counting with ^68^Ga-OncoFAP and ^68^Ga-FAPI-46 in tumor bearing mice 1 h (n=6 and n=4, respectively) (****A****) and 3 h (n=6 and n=6, respectively) (****B****) p.i.. See* ***suppl.*** ***table 2 and 3*** *for values with statistic comparisons. * indicates significance. The whisker of kidney activity for FAPI-46 are truncated in (A)* *to better display the activity range of the remainder of organs /materials.*

***
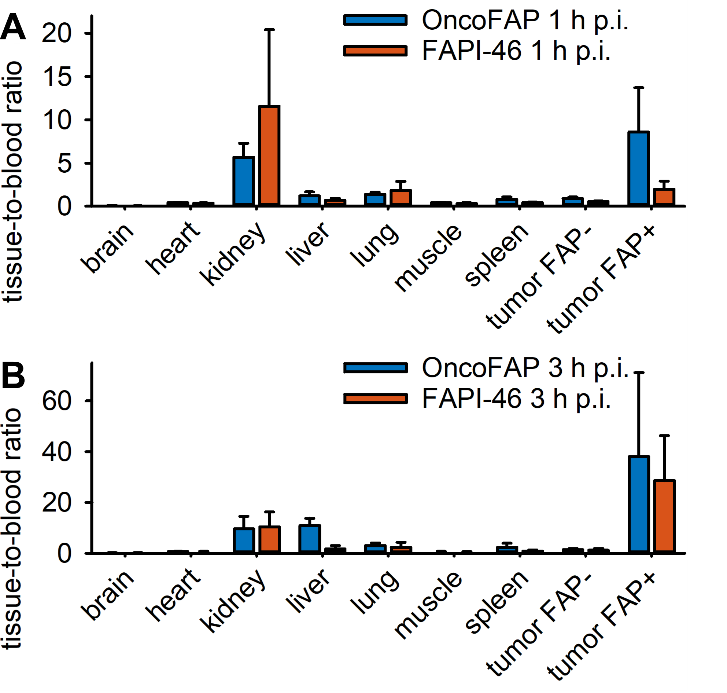
***

***Suppl. Fig. 5.*** *Tissue-to-blood ratios corresponding to Suppl. Fig. 4.*

*
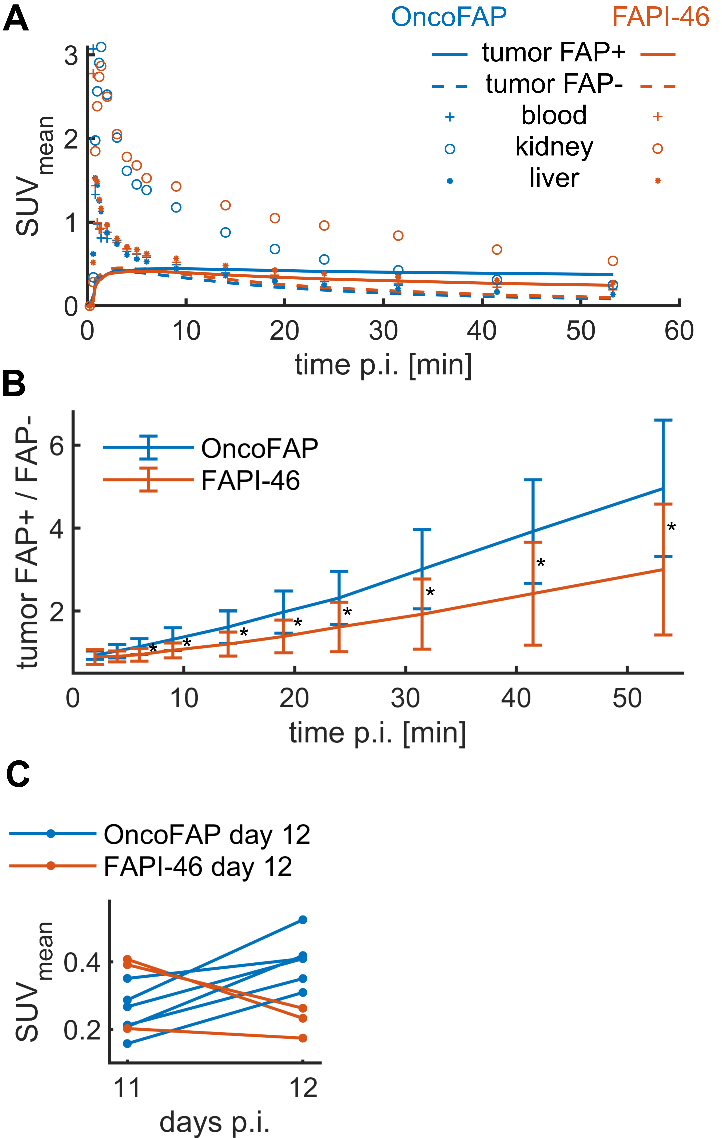
*

***Suppl Figure 6.*** *Results from head-to-head small animal PET of tumor bearing mice.* ***A*** *Mean time activity curves (TAC) of 11 mice for [^68^Ga]Ga-OncoFAP-DOTAGA and 10 mice for [^68^Ga]Ga-FAPI-46.* ***B*** *Ratio of accumulation of FAP+/FAP- tumors steadily grew over time for both tracers, but significantly stronger for [^68^Ga]Ga-OncoFAP-DOTAGA beginning after 8 minutes. * indicates significance of p<.05.* ***C****. Display of FAP+ tumor SUV_mean_ 1 h p.i. for all 9 mice that could be successfully measured at two consecutive days with both tracers. Mice measured with [^68^Ga]Ga-FAPI-46 at day 11 and with [^68^Ga]Ga-OncoFAP-DOTAGA at day 12 after implantation are highlighted in blue. Mice measured with [^68^Ga]Ga-OncoFAP-DOTAGA at day 11 and with [^68^Ga]Ga-FAPI-46 at day 12 after implantation are highlighted in red. Each of the 9 mice demonstrated stronger tracer accumulation with [^68^Ga]Ga-OncoFAP-DOTAGA irrespective of the order of applied tracers.*

*
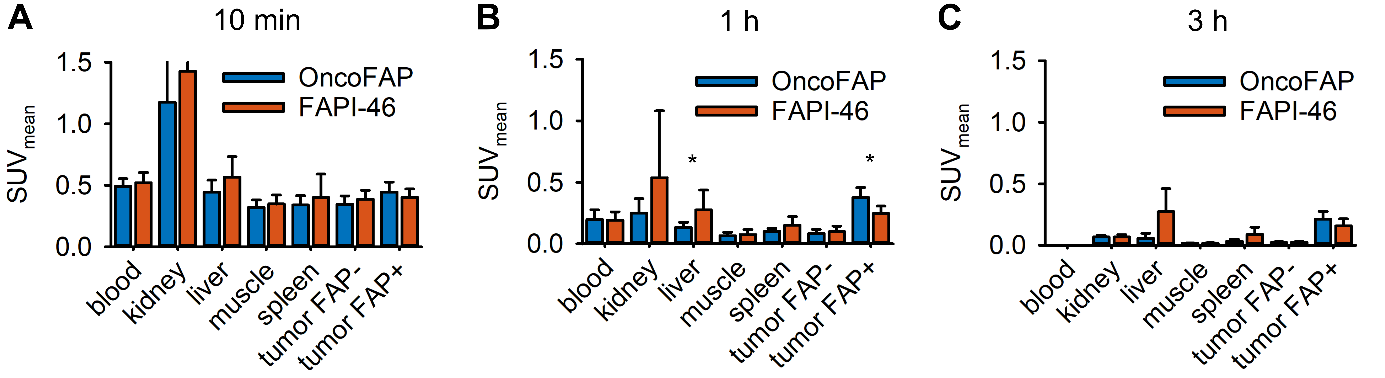
*

***Suppl Figure 7.*** *Histograms of uptake in organs and tumors 10 min* ***(A)****, 1 h* ***(B)*** *and 3 h* ***(C)*** *p.i. histograms of uptake in organs and tumors. The whiskers of kidney activity are truncated in (A) to better display the activity range of the remainder of organs /materials. * indicates significance. See* ***Suppl. Table 4*** *for values and statistical comparisons.*


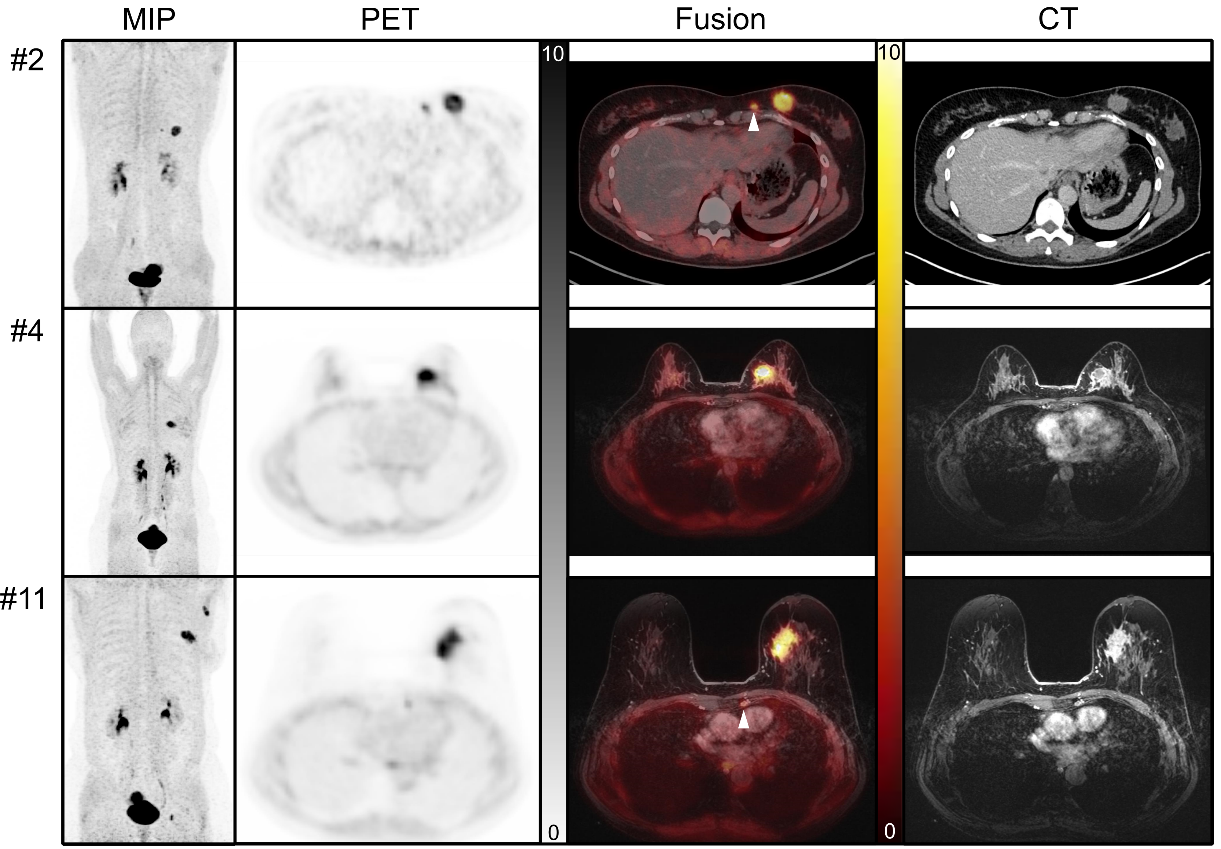


***Suppl Figure 8.*** *Images from 3 breast cancer patients.* ***Patient #2:*** *MIP, axial PET, fusion and contrast enhanced CT demonstrating avid breast tumor and small pre-pectoral lymph node metastasis (arrow).* ***Patient #4:*** *MIP, axial PET, fusion and high resolution fat saturated T1wi gadolinium enhanced MRI, avid primary breast tumor. No metastases were established in this patient.* ***Patient #11****: MIP, axial PET, fusion and high resolution fat saturated T1wi gadolinium enhanced MRI demonstrating avid tumor, axillary LN metastases and probable internal mammary lymph node metastasis (arrow).*


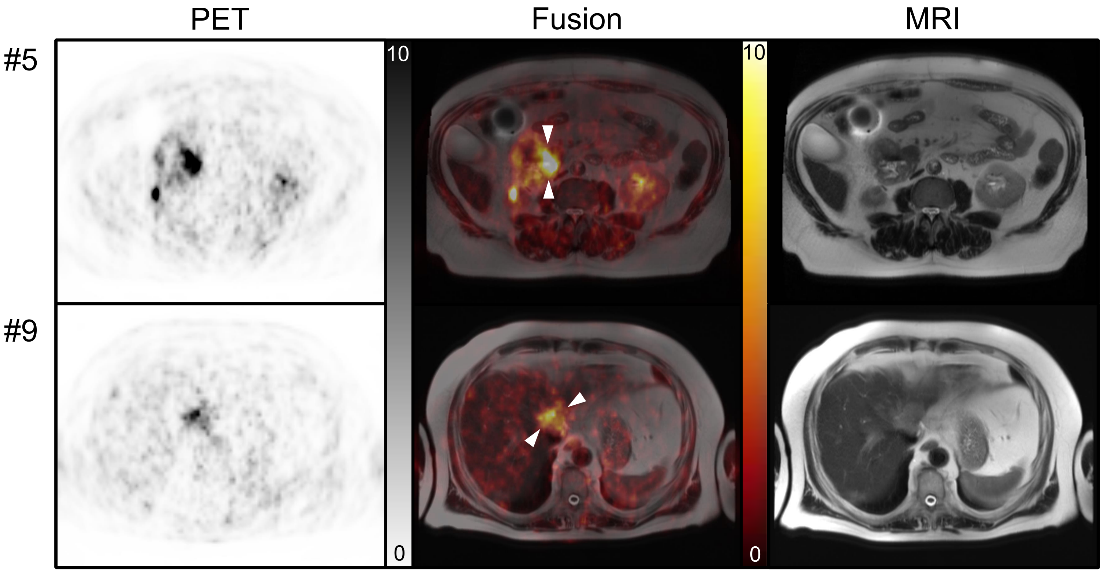


***Suppl Figure 9.*** *Images from 2 patients with abdominal tumors.* ***Patient #5:*** *Axial PET, fusion and T2wi MRI demonstrating avid tumor encasing the duodenum at the duodenal papilla (arrows). The lesion was eventually resected and classified as peritoneal metastasis of previously R1-resected right colon adenocarcinoma.* ***Patient #9****: Axial PET, fusion and T2wi MRI demonstrating avid livers tumors establishing transplant liver relapse of hepatocellular carcinoma (arrow).*
